# Higher maternal adiposity reduces offspring birthweight if associated with a metabolically favourable profile

**DOI:** 10.1007/s00125-021-05570-9

**Published:** 2021-09-20

**Authors:** William D. Thompson, Robin N. Beaumont, Alan Kuang, Nicole M. Warrington, Yingjie Ji, Jessica Tyrrell, Andrew R. Wood, Denise M. Scholtens, Bridget A. Knight, David M. Evans, William L. Lowe, Gillian Santorelli, Rafaq Azad, Dan Mason, Andrew T. Hattersley, Timothy M. Frayling, Hanieh Yaghootkar, Maria Carolina Borges, Deborah A. Lawlor, Rachel M. Freathy

**Affiliations:** 1 Institute of Biomedical and Clinical Science, College of Medicine and Health, University of Exeter, Exeter, UK; 2 MRC Integrative Epidemiology Unit, University of Bristol, Bristol, UK; 3 Department of Preventive Medicine, Northwestern University Feinberg School of Medicine, Chicago, IL, USA; 4 University of Queensland Diamantina Institute, University of Queensland, Brisbane, QLD, Australia; 5 K.G. Jebsen Center for Genetic Epidemiology, Department of Public Health and Nursing, NTNU, Norwegian University of Science and Technology, Trondheim, Norway; 6 Department of Medicine, Northwestern University Feinberg School of Medicine, Chicago, IL, USA; 7 Bradford Institute for Health Research, Bradford Royal Infirmary, Bradford, UK; 8 Department of Biochemistry, Bradford Royal Infirmary, Bradford, UK; 9 Population Health Sciences, Bristol Medical School, University of Bristol, Bristol, UK; 10 Bristol NIHR Biomedical Research Centre, Bristol, UK

**Keywords:** Adiposity, ALSPAC, BiB, BMI, EFSOCH, Glucose, HAPO, Insulin, Mendelian randomisation, UKB

## Abstract

**Aims/hypothesis:**

Higher maternal BMI during pregnancy is associated with higher offspring birthweight, but it is not known whether this is solely the result of adverse metabolic consequences of higher maternal adiposity, such as maternal insulin resistance and fetal exposure to higher glucose levels, or whether there is any effect of raised adiposity through non-metabolic (e.g. mechanical) factors. We aimed to use genetic variants known to predispose to higher adiposity, coupled with a favourable metabolic profile, in a Mendelian randomisation (MR) study comparing the effect of maternal ‘metabolically favourable adiposity’ on offspring birthweight with the effect of maternal general adiposity (as indexed by BMI).

**Methods:**

To test the causal effects of maternal metabolically favourable adiposity or general adiposity on offspring birthweight, we performed two-sample MR. We used variants identified in large, published genetic-association studies as being associated with either higher adiposity and a favourable metabolic profile, or higher BMI (*n* = 442,278 and *n* = 322,154 for metabolically favourable adiposity and BMI, respectively). We then extracted data on the metabolically favourable adiposity and BMI variants from a large, published genetic-association study of maternal genotype and offspring birthweight controlling for fetal genetic effects (*n* = 406,063 with maternal and/or fetal genotype effect estimates). We used several sensitivity analyses to test the reliability of the results. As secondary analyses, we used data from four cohorts (total *n* = 9323 mother-child pairs) to test the effects of maternal metabolically favourable adiposity or BMI on maternal gestational glucose, anthropometric components of birthweight and cord-blood biomarkers.

**Results:**

Higher maternal adiposity with a favourable metabolic profile was associated with lower offspring birthweight (−94 [95% CI −150, −38] g per 1 SD [6.5%] higher maternal metabolically favourable adiposity, *p* = 0.001). By contrast, higher maternal BMI was associated with higher offspring birthweight (35 [95% CI 16, 53] g per 1 SD [4 kg/m^2^] higher maternal BMI, *p* = 0.0002). Sensitivity analyses were broadly consistent with the main results. There was evidence of outlier SNPs for both exposures; their removal slightly strengthened the metabolically favourable adiposity estimate and made no difference to the BMI estimate. Our secondary analyses found evidence to suggest that a higher maternal metabolically favourable adiposity decreases pregnancy fasting glucose levels while a higher maternal BMI increases them. The effects on neonatal anthropometric traits were consistent with the overall effect on birthweight but the smaller sample sizes for these analyses meant that the effects were imprecisely estimated. We also found evidence to suggest that higher maternal metabolically favourable adiposity decreases cord-blood leptin while higher maternal BMI increases it.

**Conclusions/interpretation:**

Our results show that higher adiposity in mothers does not necessarily lead to higher offspring birthweight. Higher maternal adiposity can lead to lower offspring birthweight if accompanied by a favourable metabolic profile.

## Abbreviations

ALSPAC: Avon Longitudinal Study of Parents and Children
BiB: Born in Bradford
d.f: Degrees of freedom
EFSOCH: Exeter Family Study of Childhood Health
EGG: Early Growth Genetics
GWAS: Genome-wide association studies
HAPO: Hyperglycemia and Adverse Pregnancy Outcomes
IVW: Inverse variance weighted
MAGIC: Meta-Analysis of Glucose and Insulin-related traits Consortium
MR: Mendelian randomisation
SEM: Structural equation modelling
UKB: UK Biobank
WLM: Weighted linear model

## Introduction

Higher maternal BMI, an index of general adiposity, is strongly associated with higher offspring birthweight [1]. Mendelian randomisation (MR) studies support these associations as causal [2, 3]. High birthweight is associated with adverse perinatal and neonatal outcomes [4].

A likely mechanism for the association of higher maternal BMI with higher offspring birthweight is via its adverse metabolic consequences. For example, higher maternal BMI results in greater maternal insulin resistance and consequently higher maternal circulating glucose levels. As glucose crosses the placenta via facilitated diffusion, this results in increased insulin secretion by the fetus (maternal insulin cannot cross the placenta [5]). Insulin acts as a growth factor (i.e. higher fetal insulin secretion leads to increased fetal skeletal growth and fat deposition), resulting in higher birthweight [6].

Common genetic variants provide a useful tool to investigate the relationship between maternal adiposity and offspring birthweight. Genetic variants have been identified where one allele is associated with higher insulin resistance and with distributions of metabolic and adiposity traits that are consistent with a ‘metabolically obese’ phenotype (i.e. higher triacylglycerols and higher visceral/subcutaneous adiposity ratio) [7]. These associations are consistent with the ‘tissue expandability’ hypothesis, which states that population-level differences in the association between adiposity and type 2 diabetes are due to differences in the ability of adipocytes to absorb additional fatty acids from circulation, and people with either fewer adipocytes or adipocytes that are less able to increase in size (i.e. less ‘expandable’ adipose tissue) store greater fat in the visceral organs, leading to insulin resistance. The opposite allele at each of the identified genetic variants is associated with a ‘metabolically favourable adiposity’ phenotype linked to higher body fat percentage and a lower risk of type 2 diabetes [8]. In an expanded set of such variants, the metabolically favourable adiposity alleles were also associated with higher subcutaneous adipose tissue and lower ectopic liver fat [9].

It is unknown whether the metabolically favourable adiposity alleles in pregnant women affect offspring birthweight. Lack of a positive association between maternal metabolically favourable adiposity alleles and offspring birthweight would be compatible with the hypothesis that the effect of maternal BMI on birthweight is driven by the metabolic consequences of general adiposity and not by adiposity per se.

Our aim was to determine the effect of metabolically favourable adiposity on birthweight and to compare this with the effect of maternal general adiposity on birthweight. In our primary analysis, we used alleles associated with higher maternal metabolically favourable adiposity as genetic instruments to measure the effect of maternal adiposity on birthweight when coupled with ‘favourable’ metabolic effects, using data from large genome-wide association studies (GWAS) [2, 9]. We hypothesised that higher maternal metabolically favourable adiposity would either not associate with birthweight or would associate with lower birthweight if it resulted in lower maternal circulating glucose levels. In a secondary (exploratory) study, we used available individual-level data on mothers and babies to explore potential effects of maternal favourable vs general adiposity on birthweight-related metabolic (e.g. maternal glucose, cord insulin) and anthropometric (e.g. head circumference, skinfold thickness) traits.

## Methods

To estimate the causal effect of maternal metabolically favourable adiposity or BMI (exposures) on offspring birthweight (outcome), we used two-sample MR with GWAS summary statistics. In this method, estimates of SNP-exposure associations are first obtained for a set of SNPs that are robustly associated with the exposure (metabolically favourable adiposity or BMI). Second, associations between the same SNPs and outcome (birthweight) are extracted from existing GWAS datasets. For each SNP, the SNP–outcome association is then divided by the SNP–exposure association. These ratios are then pooled to give an estimate of the causative effect of the exposure on the outcome.

**Fig. 1:**
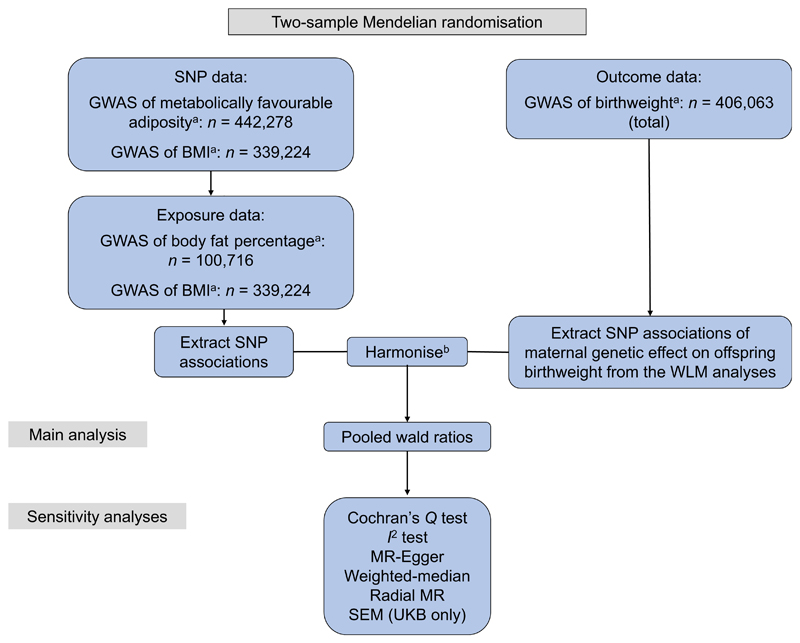
Diagram summarising the key data sources and analysis steps for the primary analyses. ^a^The GWAS data used came from Ji et al. [9] (favourable adiposity), Locke et al. [11] (BMI), Lu et al. [10] (body fat percentage) and Warrington et al. [2] (birthweight). ^b^The SNP associations were harmonised to the exposure-increasing allele [34]

The study design and different data sources are summarised in Fig. 1 and electronic supplemental material (ESM) Fig. 1. Ethical approval and informed consent from participants were obtained in all of the studies included in this research.

### Data sources

#### Genetic predictors of metabolically favourable adiposity

Metabolically favourable adiposity SNPs were identified from the most recent GWAS (*N* = 442,278) [9]. The GWAS of metabolically favourable adiposity uses a composite phenotype characterised by increased body fat percentage and a metabolic profile related to a lower risk of type 2 diabetes, hypertension and heart disease (see ESM Methods: Deriving metabolically favourable adiposity phenotype and genetic variants, and Ji et al. [9] for more details); 14 SNPs associated with higher body fat percentage and a ‘favourable’ metabolic profile were identified at *p* < 5 × 10^-8^ and replicated [9]. To facilitate the interpretation of our results we weighted these SNPs by the effect estimates of their association with body fat percentage using the latest GWAS of body fat percentage [10].

#### Genetic predictors of BMI

We used 76 BMI SNPs as instruments for general adiposity from the most recent GWAS of BMI that did not include the UK Biobank (UKB) sample (*N* = 322,154), with their weights being extracted from the same GWAS [11]. We did not use the more recent GWAS that did include UKB [12], as that would have resulted in an overlap between sample 1 (genetic instruments-BMI) and sample 2 (genetic instruments-birthweight), which could result in overfitting of the data, and bias towards confounded associations, in the presence of weak instruments [13]. Additional details of the metabolically favourable adiposity and BMI GWAS are provided in ESM Table 1 and the characteristics of the SNPs used in our MR analyses are shown in ESM Table 2.

#### Genetic associations with offspring birthweight

We used the latest GWAS of offspring birthweight from the Early Growth Genetics (EGG) consortium. A total of 406,063 participants contributed to the weighted linear model (WLM) analyses (see below), of which 101,541 were UKB participants who reported their own birthweight and birthweight of their first child, 195,815 were UKB and EGG participants with their own birthweight data, and 108,707 were UKB and EGG participants with offspring birthweight data. Further details are given in Table 1, and ESM Methods: Study Descriptions and ESM Methods: Defining offspring birthweight for GWAS (which also includes details of participant consent and ethics approval) [2].

Birthweight was standardised within UKB and each of the EGG cohorts so that SNP effect sizes are in SD units (1 SD of birthweight ≈484 g, the median SD for birthweight in 18 studies in an early birthweight GWAS [14]).

#### Genetic associations with other outcomes

Four birth cohorts were used to perform secondary analyses on birth anthropometric and cord-blood outcomes, including the Avon Longitudinal Study of Parents and Children (ALSPAC) [15, 16], Born in Bradford (BiB) [17], the Exeter Family Study of Childhood Health (EFSOCH) [18] and Hyperglycemia and Adverse Pregnancy Outcome (HAPO) study [19] (maximum *N* = 9323 mother–child pairs).

We used published GWAS summary statistics from the Meta-Analyses of Glucose and Insulin-related traits Consortium (MAGIC) to investigate the effects of the metabolically favourable adiposity and BMI SNPs on fasting glucose. The MAGIC consortium GWAS reported data from 46,186 White European adults (including men and non-pregnant women) on fasting glucose from 17 population cohorts and four case-control studies in the discovery dataset (there were 122,743 adults in the total dataset) [20]. We checked the consistency of these associations with those for pregnancy fasting glucose in BiB, EFSOCH and HAPO (pregnancy fasting glucose data was not available for ALSPAC).

Further information on these cohorts and their contribution to the study can be found in Table 2, ESM Table 3 and ESM Fig. 1 For the cohort descriptions and additional information on participant consent, ethics approval and data extraction, see ESM Methods: Study descriptions. For additional information on how ethnicity was defined for each study, see ESM Methods: Selecting participants of European ancestry. For additional information on genotyping, see ESM Methods: Genotyping. For additional information on phenotype assessment, see ESM Methods: Measuring cord-blood outcomes in selected birth cohorts for secondary analyses; and ESM Methods: Measuring pregnancy glucose outcomes in selected cohorts for secondary analyses.

### Data analyses

Our primary analysis was to study the effect of maternal metabolically favourable adiposity and BMI on offspring birthweight in the UKB and EGG meta-analysis. In addition, we undertook exploratory analyses on the effects of metabolically favourable adiposity and BMI on other outcomes (see Table 2 and ESM Fig. 1).

#### Adjusting for the fetal genotype

To avoid violating the third assumption of MR due to fetal genetic effects, we needed to adjust for the effects of the fetal genotype on the outcomes (see ESM Methods: Potential violation of MR assumptions by the fetal genotype).

For the primary analyses, to ensure our analyses considered only the effect of the maternal genotype and not the correlated fetal genotype, we used the maternal genetic effect on offspring birthweight that had been estimated using a WLM [2]. The WLM is a linear approximation of a structural equation modelling (SEM) technique that was developed to combine data from disparate study designs to estimate independent maternal and fetal genetic effects, equivalent to conditional analysis in mother-child pairs. The WLM/SEM method combines studies with own genotype data available in addition to own birthweight and offspring birthweight data, with data from studies with only their own birthweight or offspring birthweight.

Further details about WLM/SEM, and methods used to confirm that we obtained similar causal effect estimates with both the WLM- and SEM-adjusted summary statistics for birthweight, are given in ESM Methods: Structural equation modelling (SEM) and weighted linear modelling theory and ESM Methods: Extracting own birthweight data in UK Biobank (see also [2, 21]).

For the secondary analyses, the maternal genotype effects on offspring outcomes were directly adjusted for the fetal genotype, as both mother and offspring genotypes were available for the four cohorts used.

#### MR analysis

For the primary analyses, we performed two-sample MR using Wald ratios [22], which were calculated by dividing each SNP’s effect on offspring birthweight (maternal genetic effect estimated using WLM) by the same SNP’s effect on the exposure (maternal metabolically favourable adiposity or BMI). Standard errors were calculated by dividing the WLM/SEM-defined standard error of the SNP’s effect on offspring birthweight by each SNP’s effect on the exposure.

The resulting effect estimates from our MR analyses are reported as the mean difference in offspring birthweight per 1 (6.5%) SD higher maternal body fat percentage (1 SD of body fat percentage = 6.5%; see ESM Methods: Defining a 1 SD increase in body fat percentage) for metabolically favourable adiposity, and the mean difference in offspring birthweight per 1 SD increase in maternal BMI (1 SD of BMI = 4 kg/m^2^ [3]) for BMI.

For the secondary analyses, we used fixed effect pooled Wald ratio analysis to determine the effect of maternal metabolically favourable adiposity and BMI on offspring outcomes in each of these studies and compared the pooled result of that with the same result in our main analyses of effects on birthweight.

#### Sensitivity and additional analysis

For both the primary and secondary analyses, we performed additional sensitivity analyses to assess the validity of the genetic instrumental variables. This mostly included methods to test the assumptions of two-sample MR (such as Cochran’s *Q* and I^2^, leave-one-out analyses [23], MR-Egger [24], weighted-median [25] and Radial MR [26]). To further explore the hypothesis that glucose mediates the effect of BMI on birthweight, we performed a multivariable MR analysis [27] for adjusting the effect of BMI on birthweight. This could not be done for metabolically favourable adiposity, due to the fact that it is a composite trait in which insulin resistance is one of the defining features. Further details are provided in the following ESM Methods sections: Overview of tests to explore potential violations of two-sample Mendelian randomisation; Sensitivity analyses to explore horizontal pleiotropy and additional sources of invalid instruments; BMI SNP validation; Collider bias test; Cross exposure analyses; Testing potential confounders and mediators; Multivariable MR analyses for potential confounders and mediators; and Multivariable MR analyses for glucose mediation.

## Results

The associations between the SNPs analysed in the primary study and UKB + EGG birthweight are shown in ESM Table 4 and the association between the same SNPs and all the outcomes analysed for the secondary study (fasting glucose, pregnancy fasting glucose, pregnancy 2 h glucose, cord insulin, cord C-peptide, cord leptin, cord adiponectin, birthweight, birth length, ponderal index, head circumference, triceps skinfold thickness, subscapular skinfold thickness and sum of skinfold thickness) are shown in ESM Tables 5–7.

### Maternal metabolically favourable adiposity and maternal general adiposity, indexed by BMI, have opposite effects on offspring birthweight

**Fig. 2:**
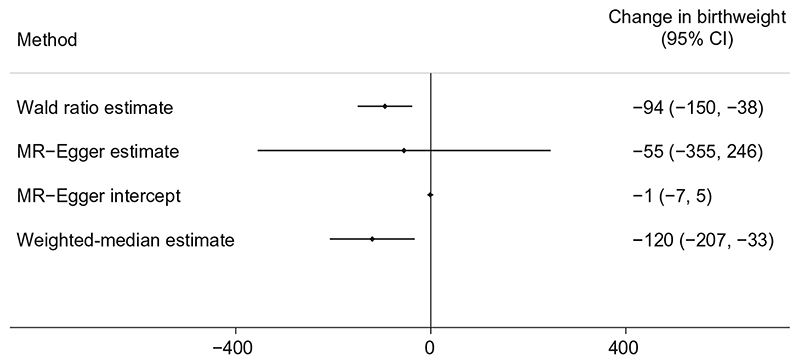
Causative effect estimates for maternal metabolically favourable adiposity on offspring birthweight. The methods (Wald ratio, MR-Egger and weighted-median analysis) used WLM-adjusted estimates to account for offspring genotype. The *x*-axis shows the change in birthweight (g) per 1 SD increase in body fat percentage (6.5%)

We found evidence that higher maternal metabolically favourable adiposity causes lower offspring birthweight (Fig. 2). The main estimate (−94 g [95% CI −150, −38]) of difference in mean birthweight per 1 SD (6.5%) higher maternal metabolically favourable body fat percentage (*p* = 0.001) was consistent with both the MR-Egger and weighted-median estimates (Fig. 2). There was evidence of heterogeneity between the Wald ratios across the SNPs (Cochran’s *Q* = 33.46 [degrees of freedom (d.f.) =13], *I* ^2^ = 61.1%, *p* = 0.001), yet results were consistent across leave-one-out analysis (ESM Fig. 2). Using the SEM method to adjust for fetal genotype effects also gave very similar results (ESM Fig. 3).

**Fig. 3:**
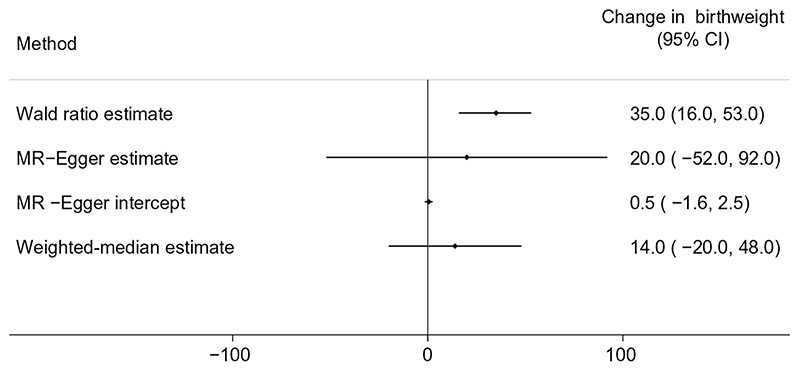
Causative effect estimates for maternal BMI on offspring birthweight. The methods (Wald ratio, MR-Egger and weighted-median analysis) used WLM-adjusted estimates to account for offspring genotype. The *x*-axis shows the change in birthweight (g) per 1 SD increase in maternal BMI (4 kg/m^2^)

The two-sample MR estimates for BMI are consistent with higher maternal general adiposity leading to higher offspring birthweight (Fig. 3). The main MR estimate was 35 g (95% CI 16, 53) of difference in mean birthweight per 1 SD (4 kg/m^2^) higher maternal BMI (*p* = 0.0002). MR-Egger (20 g [95% CI −52, 92], *p* = 0.576) and weighted-median (14 g [95% CI −20, 48], *p* = 0.424) estimates were directionally the same, though smaller than the main estimate (Fig. 3). In this analysis, there was also evidence of heterogeneity between the Wald ratios for each SNP (Cochran’s *Q* = 178.42 (d.f. = 75), *I^2^* = 58%, *p* = 2 × 10 ^−10^), yet again results were consistent across leave-one-out analysis (ESM Fig. 4). Using the SEM method to adjust for fetal genotype gave very similar results (ESM Fig. 5).

Using Radial MR, we identified four MR-Egger outlier SNPs for the metabolically favourable adiposity effect (ESM Fig. 6, ESM Fig. 7) and 18 outlier SNPs for BMI (ESM Fig. 8, ESM Fig. 9). In both cases the Radial MR results were consistent with the main results with the outliers removed.

### Secondary study results

**Fig. 4:**
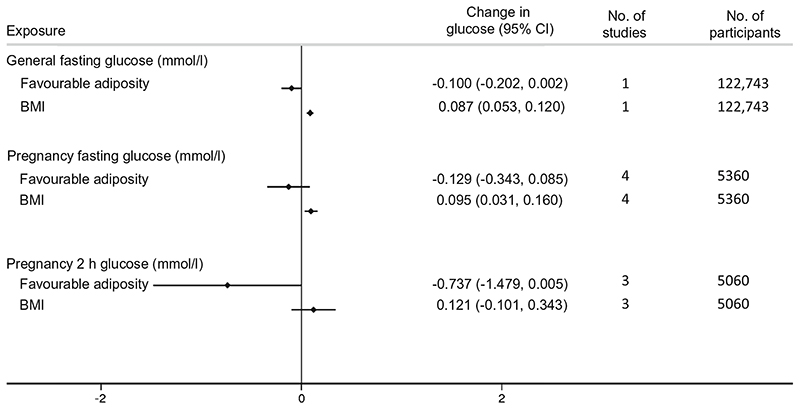
Causative effect estimates for maternal BMI and metabolically favourable adiposity on maternal pregnancy glucose outcomes. The *x*-axis shows the change in glucose outcomes per 1 SD increase in body fat percentage (6.5%) and BMI (4 kg/m^2^)

Higher metabolically favourable adiposity resulted in lower fasting glucose in men and non-pregnant women, while higher maternal BMI resulted in higher fasting glucose (Fig. 4). The relationship between maternal metabolically favourable adiposity or BMI and fasting glucose in pregnancy were consistent with this, as was 2 h postprandial glucose in pregnancy (Fig. 4). The point estimate for the effect of metabolically favourable adiposity on 2 h postprandial glucose was greater than that for fasting glucose but the CIs were wider (Fig. 4).

**Fig. 5:**
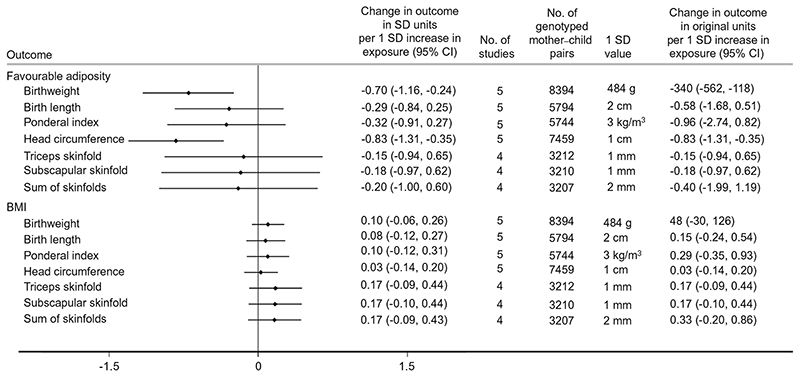
Causative effect estimates for maternal BMI and metabolically favourable adiposity on other birth anthropometric outcomes, adjusted for offspring genotype. The *x*-axis shows the change in outcomes in SDs per 1 SD increase in maternal body fat percentage (6.5%) and BMI (4 kg/m^2^)

Higher maternal metabolically favourable adiposity consistently resulted in lower neonatal anthropometric measures, in particular lower infant head circumference (Fig. 5). In contrast, evidence suggested that maternal BMI consistently resulted in higher neonatal anthropometric measures (Fig. 5).

Higher maternal metabolically favourable adiposity showed suggestive evidence of causing lower cord-blood leptin levels and, in contrast, there was strong evidence of higher maternal BMI resulting in higher cord-blood leptin levels (ESM Fig. 10). There was no detectable effect of maternal metabolically favourable adiposity or BMI on cord-blood insulin, C-peptide or adiponectin levels, though the small sample sizes made these results imprecise (ESM Fig. 10).

### Validity of the genetic instrumental variables

The BMI SNPs were positively and consistently associated with pregnancy BMI across all of the cohorts used for our secondary analyses (ESM Fig. 11).

There was no evidence that collider bias influenced the results of maternal metabolically favourable adiposity on outcomes (ESM Table 8 and ESM Fig. 12), and the effect was directionally consistent irrespective of which adiposity exposure weights were used (ESM Fig. 13, ESM Fig. 14).

The metabolically favourable adiposity genetic score was not associated with maternal smoking or maternal education level in UKB, ALSPAC, EFSOCH or BiB but it was associated with lower female educational level in HAPO (ESM Table 9). The BMI genetic score was associated with a higher prevalence of current smoking status in women in UKB and pregnancy smoking status in ALSPAC and with lower female educational level in UKB (ESM Table 9). Given these findings, we undertook further analyses using multivariable MR to see whether maternal education and smoking status confounded the association. Results from the multivariable adjusted MR analyses were consistent with those from the unadjusted MR analyses, for both smoking and years of education, adjusted for individually and when both were included in the multivariable model (ESM Table 10).

### Mediation analysis

In multivariable MR analyses, there was evidence that the effect of BMI on birthweight is mediated by its effect on fasting glucose, with the effect of BMI in the main inverse variance weighted (IVW) analyses (35 g [95% CI 6, 63] difference in mean birthweight per 1 SD increase in BMI, *p* = 0.02), attenuating with adjustment for fasting glucose (14 g [95% CI -18, 46] difference in mean birthweight per 1 SD increase in BMI, *p* = 0.39; ESM Table 11).

## Discussion

We have found evidence that higher maternal favourable adiposity lowers offspring birthweight and that higher general adiposity (BMI) increases birthweight. Our secondary analyses provided some evidence that higher maternal favourable adiposity causes lower fasting glucose in contrast to the glucose-increasing effect of higher BMI. Additional analyses of effects of maternal favourable vs general adiposity on neonatal anthropometric measures, including head circumference, were consistent in direction with those of birthweight but larger sample sizes will be needed to determine whether effect sizes varied between measures that capture fat mass (e.g. skinfold thickness) vs lean mass (e.g. head circumference). There was evidence to suggest that cord-blood leptin levels are lowered by maternal favourable adiposity and raised by higher maternal BMI. There was insufficient power to detect precise effects on cord-blood markers of fetal insulin response, as the studies used different measures of this (insulin, C-peptide or adiponectin).

Metabolically favourable adiposity is a composite of individual traits, some of which have been found to be causally associated with birthweight. In particular, higher maternal fasting glucose (often resulting from insulin resistance, a component of the metabolically unfavourable adiposity trait) has consistently been found to be causally associated with higher offspring birthweight in MR studies [2, 3]. We have shown higher metabolically favourable adiposity to be tentatively associated with lower fasting glucose, suggesting that the effect of higher maternal metabolically favourable adiposity on lower offspring birthweight may be mediated by its effect on fasting glucose levels. Though we have focused on fasting glucose, metabolically favourable adiposity is also associated with other exposures, in particular lower triacylglycerol levels [9]. However, MR studies have failed to find any evidence of an association between maternal circulating triacylglycerol levels and offspring birthweight [3, 28]. Furthermore, glucose crosses the placenta by facilitated diffusion [29] and directly influences fetal growth; for this reason, our focus in this study was on maternal fasting glucose. Our multivariable MR analysis provides some evidence that higher maternal fasting glucose has a mediating role in the effect of maternal BMI on offspring birthweight. However, it is not possible to explore this mediation with metabolically favourable adiposity because of the composite nature of this exposure and because metabolically favourable adiposity SNPs were selected based on their indirect association with glucose traits (given insulin resistance is one of the defining features of this composite trait), therefore making the results difficult to interpret (see ESM Methods: Multivariable MR analyses for glucose mediation for more details).

The metabolically favourable adiposity phenotype used here is based on the adipose ‘tissue expandability’ hypothesis. Other metabolically favourable adiposity phenotypes have been developed that focus on BMI [30], BMI and WHR [31], or BMI, WHR and body fat percentage independently [32] rather than body fat percentage alone. Unlike the metabolically favourable adiposity phenotype used in this study, those studies used correlation rather than clustering analyses. Of the two studies that reported the genetic variants they identified, the phenotype reported by Winkler et al. 2018 [31] overlapped with four of the 14 loci used in this study (*GRB14/COBLL1, VEGFA, CCDC92/DNAH10* and *FAM13A*) while Huang et al. 2021 [32] captured all but two of the loci used in this study (*CITED2* and *TRIB1/LRATD2)*. Neither of these have been used in MR studies of birthweight. The Winkler et al. 2018 [31] phenotype might not be comparable with our phenotype and could produce different results compared with this study, though the broader Huang et al. 2021 [32] phenotype might produce a similar result. Nonetheless, further studies are needed in future to test other genetic instruments for metabolically favourable adiposity phenotypes.

### Study strengths and limitations

To the best of our knowledge, this is the first study to use MR to investigate the effect of maternal metabolically favourable adiposity on offspring birthweight. We used data from a large GWAS of birthweight and for the first time examined potential effects on maternal glucose traits as well as additional newborn anthropometric and cord-blood measurements in exploratory analyses. We explored the validity of our genetic instrumental variables using multiple sensitivity analyses, including the recently developed Radial MR method [26], and found that overall results from these sensitivity analyses were consistent with our main findings. Our BMI genetic variants explained 2.7% of the variance in BMI in the original GWAS cohort [11]. As metabolically favourable adiposity is not a directly measured single trait, it is not possible to measure how much ‘variance’ is explained by the genetic variants. However, for the primary study with birthweight the MR estimates were precise.

Only 5% of those invited participated in the UKB and offspring birthweight was reported several years later. However, the similarity of the UKB results with those from the four birth cohorts with response rates of at least 70% suggests that the UKB results are unlikely to be importantly biased. Selfreport of own and first child’s birthweight and the rounding to the nearest one pound (~0.454 kg, first child’s birthweight only) may have introduced error in the birthweight measure in UKB, but this would be random with respect to genotype and would not be expected to substantially bias results. In UKB there is a relatively lower reported birthweight than in most of the other cohorts used in this study, likely reflecting secular trends of increasing birth size over time.

In UKB, genetically instrumented BMI was found to be associated with both educational attainment and smoking status. However, results of the multivariable IVW analyses of maternal BMI on offspring birthweight adjusted for smoking and years of education were not substantially different from the main result, suggesting the association was not heavily confounded (ESM Table 10).

While there was evidence of between-SNP heterogeneity of both metabolically favourable adiposity and BMI, results were directionally consistent across the main estimate, leave-one-out analyses, MR-Egger and weighted-median analyses. The Radial MR analyses for both maternal metabolically favourable adiposity and maternal BMI found evidence of SNPs with outlier effects (four for maternal metabolically favourable adiposity, 18 for maternal BMI). However, in both cases, removal of the outlier SNPs from the analyses resulted in estimates consistent with the main estimates, suggesting horizontal pleiotropy is unlikely to be a major source of bias for our analyses. In our secondary studies we had insufficient power to undertake these sensitivity analyses.

In conclusion, our results suggest that maternal metabolically favourable adiposity has the opposite effect on offspring birthweight to that of maternal BMI. This means that higher adiposity in mothers does not necessarily lead to higher offspring birthweight and may result in lower offspring birthweight if accompanied by a favourable metabolic profile. In the future, methods to stratify overweight and obese pregnant women by their metabolically favourable adiposity status could allow for targeted interventions to achieve healthy birthweight.

## Supplementary Material

The online version of this article (https://doi.org/10.1007/s00125-021-05570-9) contains peer-reviewed but unedited supplementary material.

Supplementary Information

## Figures and Tables

**Table 1:** Characteristics of studies contributing maternal genotype and offspring birthweight data to the GWAS of offspring birthweight

Characteristic	UKB	B58C-WTCCC	B58C-T1DGC	DNBC-GOYA	DNBC-PTB-CONTROL	MoBa-2008	NFBC1966	NTR	QIMR	TWINSUK	ALSPAC	EFSOCH	HAPO
Participants (*n*)	190,406	858	836	1805	1656	650	2035	707	892	1603	7304	855	1280
Country	UK	UK	UK	Denmark	Denmark	Norway	Finland	The Netherlands	Australia	UK	UK	UK	USA
Offspring year of birth	1936–1970	1972–2000	1972–2000	1996–2002	1987–2009	1999–2008	1987–2001	1946–2003	1929–1990	NA	1991–1992	2000–2004	2000–2006
Maternal age at birth of child (years)	25.3 (4.5)	26.2 (5.2)	26.1 (5.4)	29.2 (4.2)	29.9 (4.2)	28.5 (3.3)	26.5 (3.7)	27.1 (3.7)	24.5 (4.0)	NA	28.48 (4.77)	30.5 (5.9)	31.5 (5.3)
Maternal BMI (kg/m^2^)^a^	27.07 (5.03)	NA	NA	23.57 (4.27)	23.57 (4.27)	23.93 (3.94)	NA	NA	22.79 (5.13)	NA	22.93 (3.76)	24.07 (4.42)	24.5 (4.64)
Gestational age at delivery (weeks)	NA	40 (40–41)	40 (40–41)	40 (39–41)	40 (39–40)	40 (0.9)	40 (2)	40 (38–42)	NA	NA	40 (39–41)	40 (37–43)	40 (1.7)
Mothers smoking (%)	12	38	34.1	25.8	17.8	8.1	NA	NA	NA	NA	17.5	13	13.5
Birthweight (g)	3227 (477)	3325 (483)	3379 (469)	3643 (495)	3595 (497)	3679 (430)	3525 (461)	3469 (529)	3344 (532)	NA	3481 (475)	3512 (480)	3557 (517)

Data are from Warrington et al. 2019 [2] and Beaumont et al. 2018 [33] Data are shown as mean (SD) or median (interquartile range), unless otherwise stated This table only shows the studies that contributed maternal genotype and offspring birthweight data (*n =* 210,267) to the final WLM-adjusted GWAS of offspring birthweight (*n =* 406,063). More information regarding offspring genotype and own birthweight data can be found in Warrington et al. 2019 [2]

a For UKB, maternal BMI was measured at the time of study data collection and after the pregnancy for which the birthweight was reported; for the other cohorts, maternal pre-pregnancy BMI was reported B58C-T1DGC, British 1958 Birth Cohort - Type 1 Diabetes Genetics Consortium; B58C-WTCCC, British 1958 Birth Cohort - Wellcome Trust Case Control Consortium; DNBC-GOYA, Danish National Birth Cohort - Genetics of Overweight Young Adults; DNBC-PTB-CONTROL, Danish National Birth Cohort - Preterm Birth-Control Mothers; MoBa-2008, the Norwegian Mother and Baby Cohort, 2008; NA, not applicable; NFBC1966, the Northern Finland 1966 Birth Cohort; NTR, Netherlands Twin Registry; QIMR, Queensland Institute of Medical Research

**Table 2:** Characteristics of the studies used for the secondary analyses

Characteristic	ALSPAC	BiB	EFSOCH	HAPO 1^a^	HAPO 2^a^
Participants (*n*)	7411	3308	1022	1052	815
Country	UK	UK	UK	USA	USA
Offspring year of birth	1991–1993	2007–2011	2000–2004	2001–2006	2000–2006
Maternal age at birth of child (years)	28.5 (4.8)	27.1 (6.0)	30.4 (5.3)	32.1 (5.1)	29.9 (5.4)
Maternal pre-pregnancy BMI (kg/m^2^)	22.9 (3.8)	26.6 (5.9)	24 (4.4)	24.2 (4.6)	24.6 (5.3)
Gestational age at delivery (weeks)	39.6 (1.7)	39.7 (1.8)	39.9 (1.5)	40.0 (1.2)	40.0 (1.2)
Offspring sex (% male)	49.8	51.6	51.6	47.9	50.9
Mothers smoking (%)	17.2	33.1	13.3	12.9	15.1
Birthweight (g)	3495.0 (470.6)	3438.9 (481.8)	3513.2 (475.5)	3542.5 (509.1)	3539.5 (431.1)
Birth length (cm)	50.9 (2.2)	NA	50.3 (2.1)	50.5 (2.2)	51.8 (2.5)
Birth ponderal index (kg/m^3^)	26.4 (2.7)	NA	27.7 (2.6)	27.4 (3.3)	25.4 (3.3)
Birth head circumference (cm)	35.0 (1.4)	34.7 (1.4)	35.2 (1.3)	34.9 (1.6)	34.9 (1.4)
Birth triceps skinfold thickness (mm)	NA	5.2 (1.1)	4.9 (1.1)	4.1 (0.8)	4.1 (0.9)
Birth subscapular skinfold thickness (mm)	NA	4.9 (1.1)	4.9 (1.2)	4.6 (1.0)	4.3 (1.0)
Sum of birth skinfold thickness (mm)	NA	10.1 (2.1)	9.7 (2.1)	13.1 (2.5)	12.3 (2.4)
Cord-blood C-peptide (nmol/l)^b^	NA	NA	NA	0.3 (0.2–0.4)	0.3 (0.2–0.4)
Cord-blood insulin (pmol/l)^b^	NA	24.3 (15.0–41.0)	37.6 (26.0–60.0)	NA	NA
Cord-blood leptin (μg/l)^b^	NA	7.3 (4.0–13.1)	NA	NA	NA
Cord-blood adiponectin (μg/ml)^b^	NA	33.3 (26.3–42.7)	NA	NA	NA
Fasting glucose (mmol/l)	NA	4.40 (0.42)	4.35 (0.38)	4.58 (0.37)	4.51 (0.34)
2 h post-load glucose (mmol/l)	NA	5.43 (1.30)	NA	6.02 (1.20)	6.06 (1.19)

Data are presented as mean (SD) or median (interquartile range), unless otherwise stated

a For HAPO 1, genetic data was stored and analysed at the Northwestern University Feinberg School of Medicine, Chicago (IL, USA). For HAPO 2, genetic data was stored and analysed at the University of Exeter (Exeter, UK). These were non-overlapping samples of mothers and babies of European ancestry

b Since cord-blood outcomes have a non-standard distribution, the median and interquartile ranges are presented

NA, not applicable (characteristic was not measured in cohort)

## Data Availability

Our study uses two-sample MR. We used both published summary results (i.e. taking results from published research papers and websites) and individual participant cohort data: journal-published and website summary data were used for sample one of the two-sample MR (published GWAS of BMI and body fat percentage). The references to the published data sources are provided in the main paper. The data for the GWAS of BMI are available at https://portals.broadinstitute.org/collaboration/giant/index.php/GIANT_consortium_data_files. The data for the GWAS of body fat percentage are available at https://walker05.u.hpc.mssm.edu. We used individual participant data for the second MR sample and for undertaking sensitivity analyses from the UKB, ALSPAC, BiB, EFSOCH and HAPO cohorts. The data in UKB, ALSPAC and BiB are fully available, via managed systems, to any researchers. The managed system for both studies is a requirement of the study funders but access is not restricted on the basis of overlap with other applications to use the data or on the basis of peer review of the proposed science. Researchers have to pay for a dataset to be prepared for them. Full information on how to access UKB data can be found at www.ukbiobank.ac.uk/using-the-resource/. The ALSPAC data management plan (www.bristol.ac.uk/alspac/researchers/data-access/documents/alspac-data-management-plan.pdf) describes, in detail, the policy regarding data sharing, which is through a system of managed open access. The following steps highlight how to apply for access to the data included in this paper and all other ALSPAC data: (1) please read the ALSPAC access policy (PDF, 627 kB), which describes the process of accessing the data and samples in detail, and outlines the costs associated with doing so; (2) you may also find it useful to browse the fully searchable ALSPAC research proposals database, which lists all research projects that have been approved since April 2011; (3) please submit your research proposal for consideration by the ALSPAC Executive Committee and you will receive a response within 10 working days to advise you whether your proposal has been approved. If you have any questions about accessing data, please e-mail alspac-data@bristol.ac.uk. Full information on how to access BiB data can be found at https://borninbradford.nhs.uk/research/how-to-access-data/. Requests for access to the original EFSOCH dataset should be made in writing in the first instance to the EFSOCH data team via the Exeter Clinical Research Facility (crf@exeter.ac.uk).

## Research in context

### What is already known about this subject?

- Studies in non-pregnant obese people suggest that many people can have a ‘metabolically healthy’ form of obesity but effects in pregnancy and on offspring are not known
- Higher maternal BMI is causally associated with higher offspring birthweight and this may be mediated by the fetal insulin response to higher maternal gestational glucose
- Genetic variants have been identified where one allele is associated with higher adiposity but lower risk of type 2 diabetes and favourable metabolic profile, including lower insulin and glucose levels. This state is so-called ‘metabolically favourable adiposity’. The mechanism underlying this phenomenon is thought to involve greater subcutaneous adipose storage capacity, leading to higher insulin sensitivity

### What is the key question?

- What is the effect of maternal metabolically favourable adiposity on birthweight and how does it compare with the effect of maternal BMI on birthweight?

### What are the new findings?

- Across the population range, higher maternal adiposity can lead to lower, not higher, birthweight if it is also associated with a metabolically favourable profile. This contrasts with the effect of higher maternal general adiposity (proxied by BMI), which is associated with higher birthweight
- Across the population range, higher maternal metabolically favourable adiposity causes lower maternal fasting plasma glucose levels, most likely due to higher insulin sensitivity. In contrast, higher maternal general adiposity leads to higher maternal fasting plasma glucose levels, most likely due to lower insulin sensitivity

### How might this impact on clinical practice in the foreseeable future?

- Identifying ways of stratifying overweight and obese pregnant women into those with and without metabolically favourable adiposity could allow for targeted management to obtain healthy fetal growth and birthweight
