# Supplementary material for: Higher maternal adiposity reduces offspring birthweight if associated with a metabolically favourable profile
Source: Diabetologia. Author manuscript; Available in PMC 2021 Dec 1. (PMC8563674; doi:10.1007/s00125-021-05570-9)
Supplement: Supplementary Information [file EMS135428-supplement-Supplementary_Information.zip › 125_2021_5570_MOESM1_ESM.pdf]

Electronic Supplementary Material

Table of Contents

ESM Methods 3
Deriving metabolically favourable adiposity phenotype and genetic variants 3
Study Descriptions 3
UK Biobank 3
ALSPAC 3
BiB 4
EFSOCH 4
HAPO 4
Defining offspring birth weight for GWAS 5
Selecting participants of European ancestry 5
Genotyping 5
Measuring Cord-Blood outcomes in selected birth cohorts for secondary analyses 6
Measuring Pregnancy Glucose outcomes in selected cohorts for secondary analyses 7
Potential violation of MR assumptions by the fetal genotype 7
Structural Equation Modelling (SEM) and Weighted Linear Modelling theory 7
Extracting own birth weight data in UK Biobank 8
Defining a 1 SD increase in body fat percentage 9
Overview of tests to explore potential violations of two-sample Mendelian Randomisation 9
Sensitivity analyses to explore horizontal pleiotropy and additional sources of invalid instruments 9
MR-Egger 9
Weighted-Median Analysis 9
Radial MR 10
BMI SNP validation 10
Collider bias test 10
Cross exposure analyses 11
Testing potential confounders and mediators 11
Multivariable MR analyses for potential confounders and mediators 11
Multivariable MR analyses for glucose mediation 11
Tables 13
ESM Table 1: Characteristics of genome-wide association studies of the exposures 13
ESM Table 2: Details of SNPs used in our Mendelian Randomisation analyses 13
ESM Table 3: Measurement of birth anthropometric traits in selected cohorts for secondary analyses 13

ESM Table 4: Maternal SNP-birth weight associations across the included studies 13
ESM Table 5: Maternal SNP-glucose outcomes across the included studies 13
ESM Table 6: Maternal SNP-cord blood outcomes across the included studies 13
ESM Table 7: SNP-birth anthropometric outcomes for included studies 14
ESM Table 8: Association of metabolically favourable adiposity SNPs with body fat percentage, fat mass and lean mass in UK Biobank (SD units) 14
ESM Table 9: Associations between weighted allele scores and potential confounders 14
ESM Table 10: Multivariable MR for BMI's effect on birth weight in EGG + UK Biobank; confounders and mediators 14
ESM Table 11: Multivariable MR for BMI's effect on birth weight in EGG + UK Biobank; glucose 14
Figures 15
ESM Figure 1: Summary of methods and data contributing to the secondary analyses 15
ESM Figure 2: Leave-one-out analysis for the effect of maternal metabolically favourable adiposity on offspring birth weight. 16
ESM Figure 3: Comparison of Wald Ratio meta-analysis estimates calculated using WLM-adjusted SNP associations from EGG + UK Biobank with estimates calculated using SEM adjusted SNP associations from UK Biobank alone, for metabolically favourable adiposity. 17
ESM Figure 4: Leave-one-out analysis for the effect of genetically instrumented maternal BMI on offspring birth weight. 19
ESM Figure 5: Comparison of Wald Ratio meta-analysis estimates calculated using WLM-adjusted SNP associations from EGG + UK Biobank with estimates calculated using SEM adjusted SNP associations from UK Biobank alone, for BMI. 20
ESM Figure 6: Radial MR-Egger analyses with and without outliers for metabolically favourable adiposity. 22
ESM Figure 7: Radial MR graph for metabolically favourable adiposity. 24
ESM Figure 8: Radial MR-Egger analyses with and without outliers for BMI. 25
ESM Figure 9: Radial MR graph for BMI. 27
ESM Figure 10: Causative effect estimates for maternal BMI and metabolically favourable adiposity on infant cord-blood outcomes, adjusted for offspring genotype 28
ESM Figure 11: Correlations between SNP-maternal pre-pregnancy BMI associations with SNP-GWAS BMI associations for all cohorts (in kg/m^2) 29
ESM Figure 12: Genetic associations of metabolically favourable adiposity SNPs with body fat percentage, fat mass and lean mass 31
ESM Figure 13: Causative effect estimates on offspring birth weight for maternal metabolically favourable adiposity and BMI, weighted by body fat percentage 33
ESM Figure 14: Causative effect estimates on offspring birth weight for maternal metabolically favourable adiposity and BMI, weighted by BMI 35
References 37

ESM Methods

Deriving metabolically favourable adiposity phenotype and genetic variants

The metabolically favourable adiposity genetic variants were identified in a previous study[1] in three steps. In step 1 a GWAS for body fat percentage, as measured by bioimpedance, was performed in UK Biobank (N = 442,278)[1]. In step 2, a multivariate GWAS was performed combining several metabolic biomarkers together in the same multivariate analyses (i.e. body fat percentage, HDL-Cholesterol, adiponectin, sex-hormone binding globulin, triglycerides, fasting insulin and alanine transferase). In order to perform a multivariate GWAS, canonical correlation analyses were conducted as implemented by the metaCCA package in R[2]. Standard univariate GWAS analyses of quantitative traits use linear regression to estimate the linear relationship between one SNP at a time and a single outcome of interest (sometimes adjusting for covariates in the multivariable regression model). Canonical correlation analyses on the other hand involves estimating the maximum correlation between an optimally weighted linear combination of exposure variables and an optimally weighted linear combination of outcome variables. When used in the context of GWAS, this allows one to see how a given genetic variant associates with a linear combination of observed traits, in this particular instance, a combination of traits which index a metabolically favourable vs unfavourable profile. Traditionally, in order to perform multivariate GWAS using canonical correlation analyses, individual level participant data would be needed. However, the metaCCA program allows one to perform canonical correlation analyses using summary results data[2]. In step 3, SNPs that were associated at $p < 5 \times 10^{-8}$ with both body fat percentage (step 1) and with a metabolically favourable profile in the multivariate GWAS of metabolic traits (step 2) were selected. This was achieved using hierarchical clustering using the pvclust R package, a method where groups of genetic variants are clustered based on their differing associations with observed phenotypes. Finally, the genetic variants identified were then replicated in five obesity cohorts[1].

Study Descriptions

UK Biobank

Between 2006 and 2010, patients were recruited from the NHS patient registers and contacted if they lived in close proximity to one of 22 assessment centres in England, Scotland and Wales. Detailed medical data was collected on 502,655 participants, aged between 40 and 69 at recruitment [3]. A total of 190,406 women in the UK Biobank cohort who had reported their first child's birth weight were included in the primary analyses of this paper, as were 217,397 women and men who reported their own birth weight. All participants provided written informed consent, including for their collected data to be used by international scientists. UK Biobank has approval from the North West Multi-centre Research Ethics Committee (MREC), which covers the UK. UK Biobank's research ethics committee and Human Tissue Authority research tissue bank approvals mean that researchers wishing to use the resource for approved health research do not need separate ethics approval.

The UK Biobank data was pooled with EGG data for most of the analyses in this study.

ALSPAC

Women expecting a live birth between the 1st of April 1991 and 31st of December 1992 whilst living in Avon (a former county in the South-West of England centred around the city of Bristol) were invited to take part in the study. Initially 14,541 pregnancies were recruited, which resulted in 14,676 fetuses, 14,062 live births and 13,988 children alive after one year, with additional children being recruited later [4, 5]. Please note that the study website contains details of all the data that is available through a fully searchable data dictionary and variable search tool[6]. Informed consent for the use of questionnaires and clinics was obtained from the participants following the

recommendations of the ALSPAC Ethics and Law Committee at the time. Please contact the Executive at alspac-exec@bristol.ac.uk if further details are required. We used a maximum of 4,862 unrelated mother-child pairs of European ancestry with phenotype and genotype data. This cohort contributed to the analyses with the following outcomes: birth weight (included also in the EGG consortium GWAS of birth weight), birth length, birth ponderal index and birth head circumference.

BiB

Born in Bradford (BiB) is a population-based prospective pregnancy cohort that collected detailed information from 12,450 women who experienced 13,773 pregnancies. The cohort is broadly representative of the obstetric population in Bradford, a city in the North of England, in which approximately half of the births are to mothers of South Asian origin. To be eligible for BiB women had to be expected to give birth between March 2007 and December 2010 in the maternity department at Bradford Royal Infirmary. Participants were recruited primarily at their oral glucose tolerance test (OGTT) appointment, mostly between 26-28 weeks. Ethics approval was obtained for the main platform study and all of the individual sub-studies from the Bradford Research Ethics Committee[7]. We used a maximum of 1,947 unrelated mother-child pairs of European ancestry with phenotype and genotype data. This cohort contributed to the analyses with the following outcomes: birth weight, birth head circumference, birth triceps skinfold thickness, birth subscapular skinfold thickness, sum of skinfold thickness, maternal fasting glucose, maternal 2 hour post-prandial glucose levels, cord-blood insulin, cord-blood leptin and cord-blood adiponectin.

EFSOCH

Between 2000 and 2004, pregnant women from a postcode defined region of Exeter, UK and their partners were recruited via the Exeter Maternity Unit database. A total of 1,017 families (98% white European) were recruited[8], from which a total of 993 live births were included in the primary analyses of this paper. All mothers and fathers gave informed consent and ethical approval was obtained from the local review committee. We used a maximum of 674 unrelated mother-child pairs of European ancestry with phenotype and genotype data. This cohort contributed to the analyses with the following outcomes: birth weight (included also in the EGG consortium GWAS of birth weight), birth length, birth ponderal index, birth head circumference, birth triceps skinfold thickness, birth subscapular skinfold thickness, sum of skinfold thickness, maternal fasting glucose and cord-blood insulin.

HAPO

The Hyperglycaemia and Adverse Pregnancy Outcomes (HAPO) cohort recruited 28,562 pregnant women between the 1st of July 2000 and the 30th of April 2006 from 15 clinical study centres in 10 countries (United States, Canada, Barbados, United Kingdom, the Netherlands, Thailand, Israel, Australia, Hong Kong and Singapore), four of the centres being in the United States, for their oral glucose tolerance test (OGTT) between 24 and 32 weeks. In total 25,505 pregnant women underwent OGTT, however only 23,316 women were blind tested (participants were un-blinded if they showed signs of having diabetes i.e. fasting plasma glucose > 5.8 mmol/l or 2 hour glucose > 11.1 mmol/l). The protocol was approved by the institutional review board at each field centre. All participants gave written informed consent. An external data and safety monitoring committee provided oversight [9]. We used a maximum of 1,867 unrelated mother-child pairs of European ancestry with phenotype and genotype data. This cohort contributed to the analyses with the following outcomes: birth weight (included also in the EGG consortium GWAS of birth weight), birth length, birth ponderal index, birth head circumference, birth triceps skinfold thickness, birth subscapular skinfold thickness, sum of skinfold thickness, maternal fasting glucose, maternal 2 hour post-prandial glucose levels and cord-blood c-peptide.

Defining offspring birth weight for GWAS

For the EGG consortium GWAS we used for our primary analyses, 90% of the participants came from UK Biobank. In UK Biobank, multiple births and preterm births were excluded (preterm births defined as those < 2.2 kg due to a lack of gestational age information), and 90% of the birth weight values were self-reported[10].

Selecting participants of European ancestry

In UK Biobank, we defined a subset of "European" ancestry participants for inclusion in our analyses. To do this, we generated ancestry principal components (PCs) in the 1000 genomes samples. The UKB samples were then projected into this PC space using the SNP loadings obtained from the principal components analysis using the 1000 genomes samples. The UK Biobank participants' ancestry was classified using K-means clustering centred on the 3 main 1000 genomes populations (European, African, South Asian). Those clustering with the European cluster were classified as having European ancestry. The UK Biobank participants were asked to report their ethnic background. Only those reporting as either "British", "Irish", "White" or "Any other white background" were included in the clustering analysis.

For ALSPAC, we also used PCs in the 1000 genomes sample to separate out European ancestry genotyped individuals (see above).

EFSOCH only included participants of white British origin (defined using PCs) for analyses [8]. Nonetheless, principal component analysis was performed to assess ancestry of the sample using flashPCA [11]. Outliers were defined as >4.56 SD from the cluster mean (defined using 1000 Genomes European data as the reference) and excluded (n=21 individuals [0.76%])

HAPO is a multi-ethnic cohort, and ethnicity was self-reported by the participants[9].

BiB is a multi-ethnic cohort of mostly white Europeans and South Asians, and most of the participants self-reported their ethnicity. Where self-reported ethnicity was unavailable, ethnicity reported in GP records was used, and where that was unavailable, South Asians were separated from the rest of the remaining cohort using Nam Pechan[7], a computer program for identifying South Asian names[12].

Genotyping

For UKB, we analysed data from the May 2017 release of imputed genetic data (which has been extensively described elsewhere)[13], which was then pooled with the results from the EGG GWAS[10]. Given the reported technical error with non-HRC imputed variants[14], we focused exclusively on the set of ~40M imputed variants from the HRC reference panel. As we decided to use Structural Equation Modelling (SEM) as a sensitivity analyses separate from the overall UKB + EGG analyses (see Data Analyses in main paper), we also analysed the UKB participants separately.

To account for population structure and relatedness in UKB, a linear mixed model implemented in BOLT-LMM v2.3[15] was used to perform genome-wide association (GWA) analysis of birth weight in the UKB sample. Only autosomal single nucleotide polymorphisms (SNPs) which were common (MAF>1%), in Hardy Weinberg equilibrium (p value > 1x10-6), passed QC in all 106 batches and were present on both genotyping arrays were included in the genetic relationship matrix (GRM). For the GWA analyses of birth weight of the first child (i.e. using the maternal genotype), the genotyping array and genotyping release (interim vs. full) were included as covariates in the regression model. For the GWAS of participants own birth weight, genotyping array, age at baseline and sex were adjusted for in all models.

For ALSPAC, EFSOCH, HAPO and BiB, the SNPs used in this study (see below) were taken from genome-wide imputed data that had been completed for both the mothers and their offspring (fetal genotype). In ALSPAC maternal data was obtained from the Illumina 610 Quad Array and fetal data was obtained from the Illumina 550 Quad Array. In EFSOCH maternal and fetal data were obtained from the Illumina Infinium HumanCoreExome-24, and in BiB maternal and fetal data was obtained from two separate chips, an Illumina HumanCoreExome array and Illumina Infinium Global Screening array (GSA). In HAPO maternal and fetal data were obtained from Illumina genome-wide arrays at the Broad Institute (Cambridge, MA) or Johns Hopkins Center for Inherited Disease Research (Baltimore, MD)[5, 8, 16, 17].

For both ALSPAC and EFSOCH, genotype data were imputed against Haplotype Reference Consortium HRC v1.1 reference panel after quality control (MAF >1%, HWE>1×10-6, sex mismatch, kinship errors and 4.56 SD from the cluster mean of any sub-populations cluster). For HAPO, genotype data were imputed using SHAPEIT v.2 and IMPUTE2 v.2.3.0 with 1000 Genomes Phase 3 data after quality control as previously described. For BiB, genotype data were imputed against HRC r1.1 using Minimac4, after quality control (MAF >1% and HWE>1×10-6)[5, 8, 18].

For UKB + EGG[10] and MAGIC[19], the summary results of associations between SNPs and offspring birth weight (maternal genotype) was extracted from the GWAS results (see main paper for details on how each GWAS was conducted). For ALSPAC, EFSOCH, HAPO and BiB, individual level SNP data was extracted and summary data were generated using multivariate linear regression of birth weight against the maternal SNPs (adjusting for gestational age, child’s sex and genotype).

To make sure that the outcome data (birth weight) and exposure data (metabolically favourable adiposity and BMI) were comparable, the SNPs effects were harmonized to the metabolically favourable adiposity/BMI raising alleles using procedures that have previously been described[20].

Measuring Cord-Blood outcomes in selected birth cohorts for secondary analyses

In BiB, cord blood was extracted from a vein or artery by the attendant mid-wife at delivery. Samples were refrigerated at 4°C in EDTA tubes until collected by laboratory staff within 12 hours. Samples were then spun, frozen and stored at -80°C. They were transferred to the Biochemistry Department of Glasgow Royal Infirmary for analyses (with no previous thawing), where leptin and adiponectin were measured by a highly sensitive in house ELISA with better sensitivity at lower levels than commercial assays. Insulin was measured using an ultrasensitive solid-phase two-site immunoassay ELISA (Mercodia, Uppsala, Sweden) that does not cross-react with pro-insulin. Laboratory staff were blinded to the participants ethnicity and other characteristics[21].

In EFSOCH, cord blood was extracted from a vein or artery by the attendant mid-wife at delivery. The blood was stored at 4°C until being collected by the researchers. The cord blood was spun to separate out the plasma which was then stored at -80°C. The plasma was then tested for insulin levels when appropriate at the Regional Endocrine Laboratories (Birmingham, UK) using immunochemiluminometric assays (Molecular Light Technology, Cardiff, U.K.)[8, 22].

In HAPO, cord blood plasma was extracted at each centre, was stored at -20°C and sent to the Central Laboratory for analysis. A subsect of plasma was then stored at -70°C, before being tested for c-peptide using a solid-phase, two-site fluoro-immunometric assay (Autodelfia, Perkin-Elmer, Waltham, Massachusetts, United States). C-peptide has an advantage over insulin in that it is less likely to be destroyed by haemolysis, thus allowing for a more accurate representation of cord insulin levels if haemolysis has occurred in a substantial number of samples [23].

Measuring Pregnancy Glucose outcomes in selected cohorts for secondary analyses

In BiB, all women were offered a 75g oral glucose tolerance test (OGTT) at around 26-28 weeks gestation, with samples for analyses of fasting glucose and 2-hour post-prandial glucose being collected. Women attend the OGTT having fasted overnight. Samples were immediately processed, and plasma glucose concentrations (mmol/l) were measured at the clinical biochemistry laboratory of the Bradford Royal Infirmary using a Siemens Advia 2400 analyser following a standard protocol. The coefficients of variation range between 1·73% at 3·2 mmol/L and 0·64% at 19·1 mmol/L[21, 24].

In EFSOCH, fasting blood samples were taken from both parents at 28 weeks of gestation (the parents having fasted for 10 hours); this was done in the morning at the parents' home. Plasma glucose levels were measured at the Royal Devon and Exeter Hospital pathology laboratories using manufacturer's standard reagents on Modular analysers (Roche Diagnostics, Lewes, East Sussex, U.K.)[8, 25].

In HAPO, women underwent a 75g OGTT between 24 and 32 weeks gestation, trying to be as close to 28 weeks as possible, with samples for analyses of fasting glucose and 2-hour post-prandial glucose being collected. Aliquots of plasma glucose samples were sent to field centre laboratories to be analysed. Values were un-blinded if fasting plasma glucose (FPG) was >5.8 mmol/L, if 2-h OGTT plasma glucose (PG) was >11.1 mmol/L. Otherwise, women, caregivers, and HAPO Study staff (except for laboratory personnel) remained blinded to glucose values. All glucose samples were additionally analysed at the HAPO Central Laboratory (Belfast, Northern Ireland, U.K.) using a chemical analyser (Vitros 750; Ortho Clinical Diagnostics, Rochester, NY), in order to avoid bias from centre-to-centre variation. Only the results from women who remained blinded were included in the analyses[26].

Individual studies in MAGIC, measured fasting glucose from whole blood, plasma or serum (or a combination of all three) with whole blood fasting glucose being corrected to plasma fasting glucose by multiplying the result by 1.13. Participants were excluded if they were diagnosed with diabetes, undergoing diabetes treatment, had a fasting glucose level greater than 7 mmol/l, pregnant, did not fast or had fasting glucose or fasting insulin greater than three standard deviations away from the mean[19].

Potential violation of MR assumptions by the fetal genotype

The third MR assumption is that the genetic instrument only influence the outcome through the exposure. Violation of this assumption may occur through horizontal pleiotropy and we describe below the sensitivity analyses that we used to explore that in our main analyses. A further potential violation of this assumption in this study is a path via the fetal genotype[27]. Maternal genetic variants that influence BMI and/or favourable adiposity will also be associated with the distribution of said genetic variants in the fetus, which may influence fetal growth and hence birth weight. For the primary study, this potential bias was addressed using WLM adjusted weights (see below). For the secondary study, this potential bias was addressed by adjusting the outcome-maternal SNP associations for the fetal genotype, which was available in in all four cohorts (ALSAPC, BiB, EFSOCH and HAPO) contributing to the secondary study.

Structural Equation Modelling (SEM) and Weighted Linear Modelling theory

SEM can be used to estimate the maternal specific genetic effect on offspring birth weight conditional on the offspring genotype in the absence of genetic data in mother-child pairs[28]. The model uses the participant's genotype, own birth weight measurement and their offspring's birth weight as observed variables. For each individual, these observed variables are combined with two latent (unobserved) variables, the individual's mother's genotype and the individual's offspring's

genotype, which are correlated 0.5 with the participants own genotype. A full description of SEM can be found in Warrington et al 2018[28]. In brief, the model uses the variances and co-variances between the observed variables (own birth weight, offspring birth weight and own genotype), to estimate the parameters of interest including that maternal and fetal specific effects. The model is flexible in that it can incorporate a subsect of participants with only their own birth weight and own genotype (including males) or their own genotype and offspring birth weight. However, fitting the model using full information maximum likelihood is computationally intensive. For this reason, the authors developed a linear approximation of the SEM that yielded similar effect estimates and standard errors but was more computationally efficient. This linear approximation, referred to as the weighted linear model (WLM), combined unadjusted maternal and fetal effect estimates at a single locus, using the following formula

$$\hat{\beta}_{m_{adj}} = \frac{4}{3}\hat{\beta}_{m_{unadj}} - \frac{2}{3}\hat{\beta}_{f_{unadj}}$$

where $\hat{\beta}_{m_{adj}}$ is the estimated maternal genetic effect on the outcome (adjusted for fetal genotype), $\hat{\beta}_{m_{unadj}}$ is the unadjusted maternal genetic effect from an unconditional GWAS of maternal genotype on offspring birth weight and $\hat{\beta}_{f_{unadj}}$ is the unadjusted fetal genetic effect from an unconditional GWAS of own genotype on own birth weight[10, 28].

Standard errors for the maternal genetic effect (adjusted for fetal genotype) can be calculated, assuming no overlapping individuals across the different samples, using the following formula.

$$SE\left(\hat{\beta}_{m_{adj}}\right) = \sqrt{\left(\frac{16}{9}var\left(\hat{\beta}_{m_{unadj}}\right) + \frac{4}{9}var(\hat{\beta}_{f_{unadj}})\right)}$$

The estimated maternal genetic effect from the WLM, $\hat{\beta}_{m_{adj}}$, is asymptotically unbiased and similar to the estimated effect from a conditional linear model in mother-child pairs, where offspring birth weight is regressed on the maternal and offspring genotype[10].

To confirm we obtained similar causal effect estimates with both the WLM and SEM adjusted summary statistics for birth weight, we applied the SEM method to obtain the maternal specific genetic effect on offspring birth weight at each of the SNPs, adjusted for the fetal genotype, using UKB participants only (own birth weight N = 211,815; offspring birth weight N = 187,120) and repeated the main MR analysis.

Extracting own birth weight data in UK Biobank

In order to perform the SEM in UK Biobank, we needed data on participants' own birth weight, in addition to the data on offspring birth weight (described above). A total of 280,315 participants reported their own birth weight in kilograms at either the baseline visit or at least one of the follow-up visits. Participants reporting being part of a multiple birth were excluded from our analyses (N=10,057). For participants reporting birth weight at more than one visit (N=11,629), the mean across the reported birth weights were used, and if the largest difference between any 2 time points was >1kg, they were excluded (N=80). Data on gestational duration were not available. However, in order to exclude likely pre-term births, participants with birth weight values <2.5kg were excluded. This only reduced the population sample by 5%, which is unlikely to introduce collider bias. We also excluded those with a birth weight >4.5kg as these are likely to be reporting errors or extreme outliers (total number excluded because of <2.5kg or >4.5kg birth weight=37,691). Participants' own

birth weight was regressed against year of birth and assessment centre location. Residuals from that regression model were then used in all analyses with values converted to standard deviation units for analysis.

Defining a 1 SD increase in body fat percentage

In the original GWAS of body fat percentage, the strongest SNP was associated with a 0.051 SD increase in body fat percentage, which was equivalent to a 0.33% increase in body fat[29], hence dividing 0.33 by 0.051 we estimated the SD of body fat percentage to be 6.5%. The SD value of BMI in UKB has previously been reported to be 4 kg/m2[30].

Overview of tests to explore potential violations of two-sample Mendelian Randomisation

Standard MR methods, such as fixed effect pooled Wald ratios, assume that genetic influences i) are robustly (replicated) and statistically related to the exposure (i.e. metabolically favourable adiposity and BMI), ii) are not related to confounders of exposure on the outcome and iii) only influence the outcome (i.e. birth weight) through the exposure. The third assumption can be violated by horizontal pleiotropy, and between SNP Wald ratio heterogeneity is indicative of that. Therefore we used Cochran's Q test, I2 and leave-one-out analysis to explore between SNP heterogeneity[31]. We also used MR-Egger[32], weighted-median estimator[33] and Radial MR[34] as sensitivity analyses to explore the extent that horizontal pleiotropy may have biased the results (see ESM Methods for further details of these methods and their assumptions). This sensitivity analyses was only performed for the primary analyses.

Sensitivity analyses to explore horizontal pleiotropy and additional sources of invalid instruments

MR-Egger

MR-Egger uses linear regression of the SNP associations with birth weight against the SNP associations with maternal metabolically favourable adiposity or BMI, but does not force the intercept through zero, thus relaxing the assumption that the SNP influences birth weight only through maternal metabolically favourable adiposity or BMI [32]. If a non-zero intercept is observed, this indicates that there may be bias in the fixed effect pooled Wald Ratios and/or inverse variance weighted (IVW) instrumental variable estimates due to horizontal pleiotropy. Whilst relaxing the no horizontal pleiotropy assumption and providing an estimate that takes account of non-symmetrical pleiotropy (the slope value), MR-Egger, and for that matter all analyses we have used to test for horizontal pleiotropy, introduces an additional assumption - the Instrument Strength Independent of Direct Effect (INSIDE) assumption. INSIDE assumes that the association of the genetic instrument with the exposure is not correlated with the association of the genetic instrument with the outcome (i.e. the association with outcome that is not via the exposure of interest). In relation to this study the INSIDE assumption is likely to be violated via offspring genotype because of the association of maternal genotype to risk factor and to her offspring genotype[27], and so MR-Egger is unlikely to be a useful approach for testing this source of bias. We used WLM, SEM and mother-child pair analyses to adjust for this (see methods in main paper) and used MR-Egger as a sensitivity analysis to explore possible violation of the exclusion restriction criteria via maternal genetic horizontal pleiotropy. For our MR-Egger analyses we estimated the standard error using a random effects model and confidence intervals using a t-distribution.

Weighted-Median Analysis

With weighted-median analysis, the weighted-median instrumental variable of all the SNPs is taken as the causative effect, with each SNP being weighted by its effect on the exposure, thus reducing the effect of single weak instruments[33]. This method also relaxes the assumption of there being no

bias due to asymmetrical horizontal pleiotropy but it assumes that no more than 50% of the combined SNPs weight is from invalid instruments. This approach will be biased if there is a single horizontal pleiotropic SNP with 50% of the weight or multiple pleiotropic SNPs, each with less than 50% of the weight, but that together are 50% or more of the weight. As with MR-Egger this is likely to be violated by offspring genotype as 50% of maternal alleles will be transferred to the fetus; our fetal genotype adjusted results are the key way of testing for bias via that route[27]. The weighted median analyses were as a sensitivity analysis to explore possible violation of the exclusion restriction criteria via maternal genetic horizontal pleiotropy.

Radial MR

As an additional sensitivity analyses we performed Radial MR. Radial MR is a linear regression, where the square root of the SNPs weighting (which for first-order weights is equivalent to the SNP-exposure divided by the SNP-outcome standard error) is plotted on the x-axis and the Wald ratio multiplied by the square root of the SNPs weighting is plotted on the y-axis. Radial MR has advantages over traditional MR-Egger, in that it is a better method for detecting and visualising outlying genetic instruments, and allows for more precise and accurate estimates of the intercept, making it better at detecting horizontal pleiotropy. We also performed Rückers Q test and I^2 to explore between SNP heterogeneity in the Radial MR results[34].

BMI SNP validation

The first MR assumption is that the genetic instruments are robustly associated with the exposure. The metabolically favourable adiposity and BMI genetic instruments were discovered in separate, non-overlapping GWAS of mixed sex individuals who were not pregnant, hence it is possible that the genetic instruments do not associate with adiposity in the same way in pregnancy. We therefore tested the SNPs association with BMI (only UKB had body fat percentage) in ALSPAC, EFSOCH, HAPO and BiB, and compared that to the effect reported in the main BMI GWAS. We were not able to find studies with relevant genotypes and body fat percent measured in pregnancy and so could not explore whether the genetic variants we used for the metabolically favourable adiposity related to percent body fat in a similar way in pregnancy as in the original GWAS.

The SNP associations with mothers pre-pregnancy BMI for each of the mother-child pair cohorts were pooled. The pooled SNP-maternal BMI associations were then regressed against the GWAS reported SNP-BMI associations. We also regressed the SNP-maternal BMI associations for each cohort individually.

Collider bias test

For the two sample MR that we used in the primary study, an additional risk of bias may arise from adjustments made in the exposure GWAS[35]. This may have influenced our MR analyses of metabolically favourable adiposity because body fat percentage is conditional on the total body weight and conditioning on heritable traits (such as weight) can introduce collider bias in genotype-phenotype associations[36].

For the metabolically favourable adiposity SNPs, we extracted the SNP-associations (in SD units) for body fat percentage (N=442,278), fat mass (N=442,449) and lean mass (N=443,134) from UK Biobank (see **ESM Table 8** for summary data). We then pooled the SNP-associations for each trait (unweighted) to get a per allele score for the SNPs effect on each trait. If the metabolically favourable adiposity SNPs had a stronger relative effect on lean mass than fat mass, that would indicate the presence of collider bias.

Cross exposure analyses

For the two sample MR that we used in the primary study, we weighted the metabolically favourable adiposity SNPs by their effect on body fat percentage, whilst we weighted the "general" adiposity SNPs by their effect on BMI. Whilst body fat percentage and BMI are both measures of adiposity, BMI does not distinguish between fat mass and lean mass, meaning it is not possible to directly compare them.

We therefore decided to perform cross exposure analyses, both weighting the metabolically favourable adiposity SNPs by their effect on BMI and the BMI SNPs by their effect on body fat percentage. For this, we used the same exposure data sets as used in the primary analyses (Lu et al 2016[29] for body fat percentage and Locke et al 2015[37] for BMI), and used the same Wald ratio method as the primary analyses.

Testing potential confounders and mediators

The second MR assumption is that the genetic instruments are not associated with known confounders of the relationship between the exposure and outcome (which could mediate/bias the effect estimate[38]). To explore the possible associations of SNPs with observed confounders, we calculated two weighted allele scores (WAS) from the instrumental variable SNPs for metabolically favourable adiposity and BMI and determined the "per allele" association of these WAS with maternal smoking and socioeconomic position (SEP; assessed using maternal educational attainment). Smoking is one of the strongest determinants of fetal growth and birth weight[39], and influences adiposity[40]. SEP captures (to some extent) other potential confounders, such as maternal diet, that also relate to adiposity and potentially birth weight. Each SNP was weighted by the magnitude of its effect on the exposure as reported in the original GWAS. These associations were examined in UKB, ALSPAC, BiB, EFSOCH (which does not have data on education so that was not examined in this cohort) and HAPO.

If a substantial association with a WAS and a potential confounder/mediator was found, we redid the analyses adjusting for the confounder/mediator using multivariable MR analyses (see below).

Multivariable MR analyses for potential confounders and mediators

To adjust for maternal smoking in the MR analyses of BMI's effect on birth weight, we used the only genome-wide significant SNP (rs3025343) from the Furberg et al 2010[41] GWAS of former vs current smoking. To adjust for maternal education in the multivariable MR of the effect of BMI on birth weight we aimed to use SNPs that were genome-wide significant and replicated from the most recent GWAS of completed years of education that were not discovered using UK Biobank[42]. In both analyses we used the IVW method for the multivariable MR analyses. This requires summary data on all of the: exposure SNP associations with exposure, outcome and confounder and confounder SNP associations with confounder outcome and exposure.

Multivariable MR analyses for glucose mediation

Considering the finding that metabolically favourable adiposity and BMI have opposing effects on glucose outcomes both outside and during pregnancy (see **Figure 4**), we hypothesised that the differing effects of metabolically favourable adiposity and BMI could be mediated by differing effects on plasma glucose.

To explore the glucose mediation hypotheses further, we performed a multivariable MR analyses for BMI and glucose. We decided against performing a multivariate MR analyses for metabolically favourable adiposity and glucose for two reasons. Firstly, metabolically favourable adiposity is a composite phenotype, therefore there is no appropriate summary data from which to extract the

glucose SNP and exposure association. Secondly, one of the variables used to define the metabolically favourable adiposity trait is fasting insulin, a marker of insulin resistance[1], which is known to be associated with fasting glucose levels[19]. Therefore, the metabolically favourable adiposity SNPs were selected based on their indirect association with glucose traits, rendering any glucose mediation analyses with these SNPs difficult to interpret.

To adjust for maternal fasting glucose in the MR analysis of the effect of BMI on birth weight, we used SNPs that were genome-wide significant and replicated in the MAGIC consortium GWAS of fasting glucose in the general population[19]. We used the IVW method for the multivariable MR analyses. We verified that the BMI instrument adjusted for glucose was strong enough to detect an effect: the F-statistic for the BMI instrument was 66.28, the conditional F-statistic for the BMI instrument adjusted for glucose was 50.53[43].

Tables

ESM Table 1: Characteristics of genome-wide association studies of the exposures

See ESM Tables.xlsx

a) For Locke et al 2015[37] and Lu et al 2016[29], the Recovery participants are those with Metabochip data.
b) For Ji et al 2019[1], the Recovery participants are the participants from the GWAS that contributed to the multivariate analyses.

ESM Table 2: Details of SNPs used in our Mendelian Randomisation analyses

See ESM Tables.xlsx

ESM Table 3: Measurement of birth anthropometric traits in selected cohorts for secondary analyses

See ESM Tables.xlsx

ESM Table 4: Maternal SNP-birth weight associations across the included studies

See ESM Tables.xlsx

ESM Table 5: Maternal SNP-glucose outcomes across the included studies

See ESM Tables.xlsx

ESM Table 6: Maternal SNP-cord blood outcomes across the included studies

See ESM Tables.xlsx

a) In order to compare the cord-blood outcomes with each other, and owing to the fact that insulin has a non-standard distribution, the cord-blood outcomes were logged for analyses. The original units, some of which were not SI units, were pmol/l for insulin, µg/L for C-peptide, µg/mL for adiponectin and ng/mL for Leptin.

ESM Table 7: SNP-birth anthropometric outcomes for included studies

See ESM Tables.xlsx

ESM Table 8: Association of metabolically favourable adiposity SNPs with body fat percentage, fat mass and lean mass in UK Biobank (SD units)

See ESM Tables.xlsx

ESM Table 9: Associations between weighted allele scores and potential confounders

See ESM Tables.xlsx

ESM Table 10: Multivariable MR for BMI's effect on birth weight in EGG + UK Biobank; confounders and mediators

See ESM Tables.xlsx

a) For the education adjustment, of the 74 non-UK Biobank identified SNPs from Okbay et al[42], only 33 had both birth weight data in EGG + UK Biobank data and BMI data in Locke et al[37], and only five had smoking data in Furberg et al[41] as well.

ESM Table 11: Multivariable MR for BMI's effect on birth weight in EGG + UK Biobank; glucose

See ESM Tables.xlsx

a) For the glucose adjustment, we used 16 SNPs identified from Dupuis et al 2010[19]
b) Adjusting for fasting glucose attenuated the effect of BMI. The unadjusted result was 35g (95% CI, 6 to 63) per 1 SD increase in BMI, whilst the adjusted result was 14g(95% CI, -18 to 46) per 1 SD increase in BMI. The F-statistic for both the unadjusted and adjusted BMI instrument was above 10, hence substantial bias due to weak instruments is unlikely. Therefore, it seems much of the effect of BMI on birth weight is mediated by glucose.

Figures

ESM Figure 1: Summary of methods and data contributing to the secondary analyses

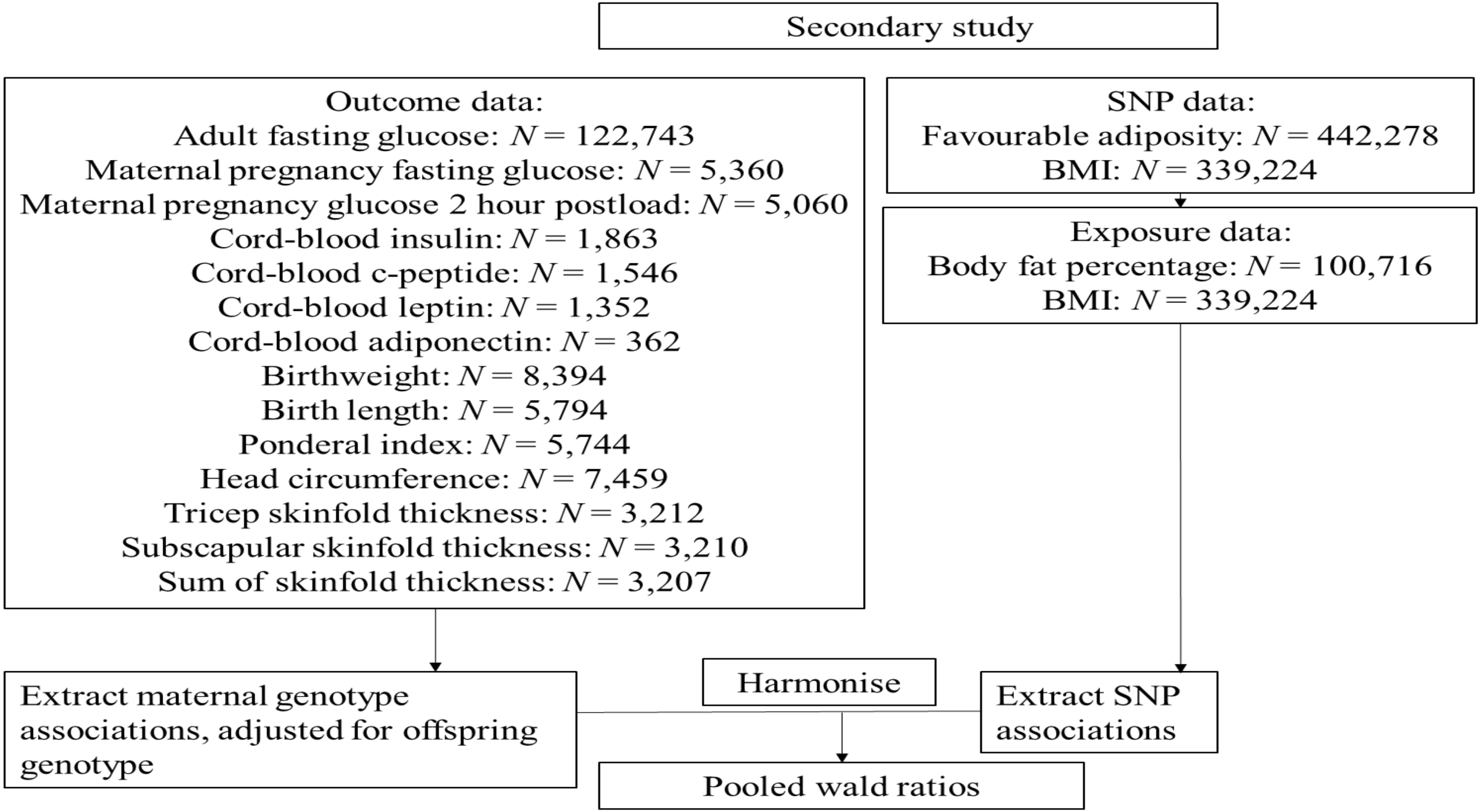

ESM Figure 2: Leave-one-out analysis for the effect of maternal metabolically favourable adiposity on offspring birth weight.

The x-axis shows the effect on birth weight (grams) per 1 SD increase in body fat percentage (6.5%)

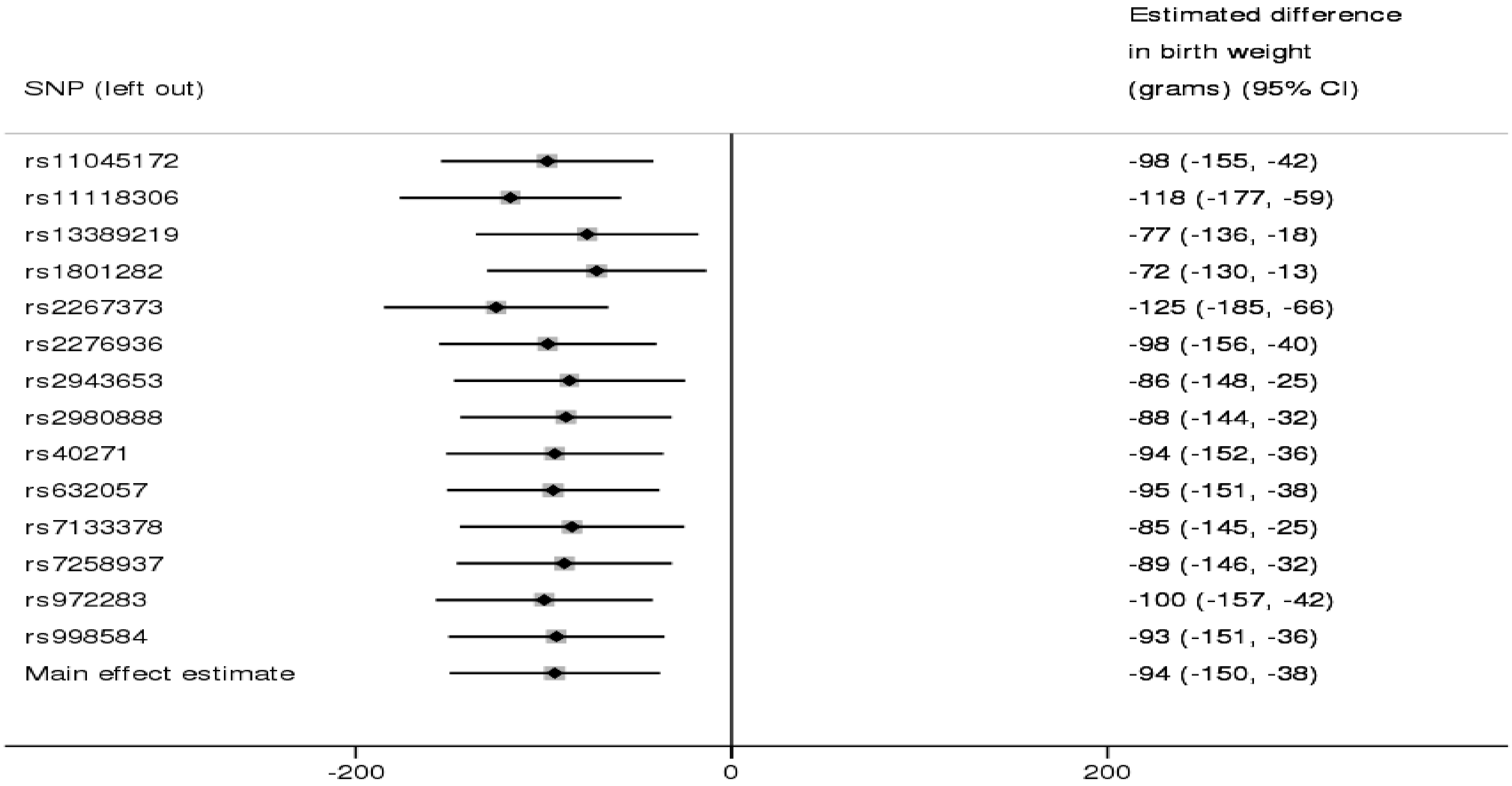

ESM Figure 3: Comparison of Wald Ratio meta-analysis estimates calculated using WLM-adjusted SNP associations from EGG + UK Biobank with estimates calculated using SEM adjusted SNP associations from UK Biobank alone, for metabolically favourable adiposity.

The x-axis shows the change in birth weight (g) per 1 SD increase in body fat percentage (6.5%), and the ES shows the estimated difference in birth weight (g) for each method.

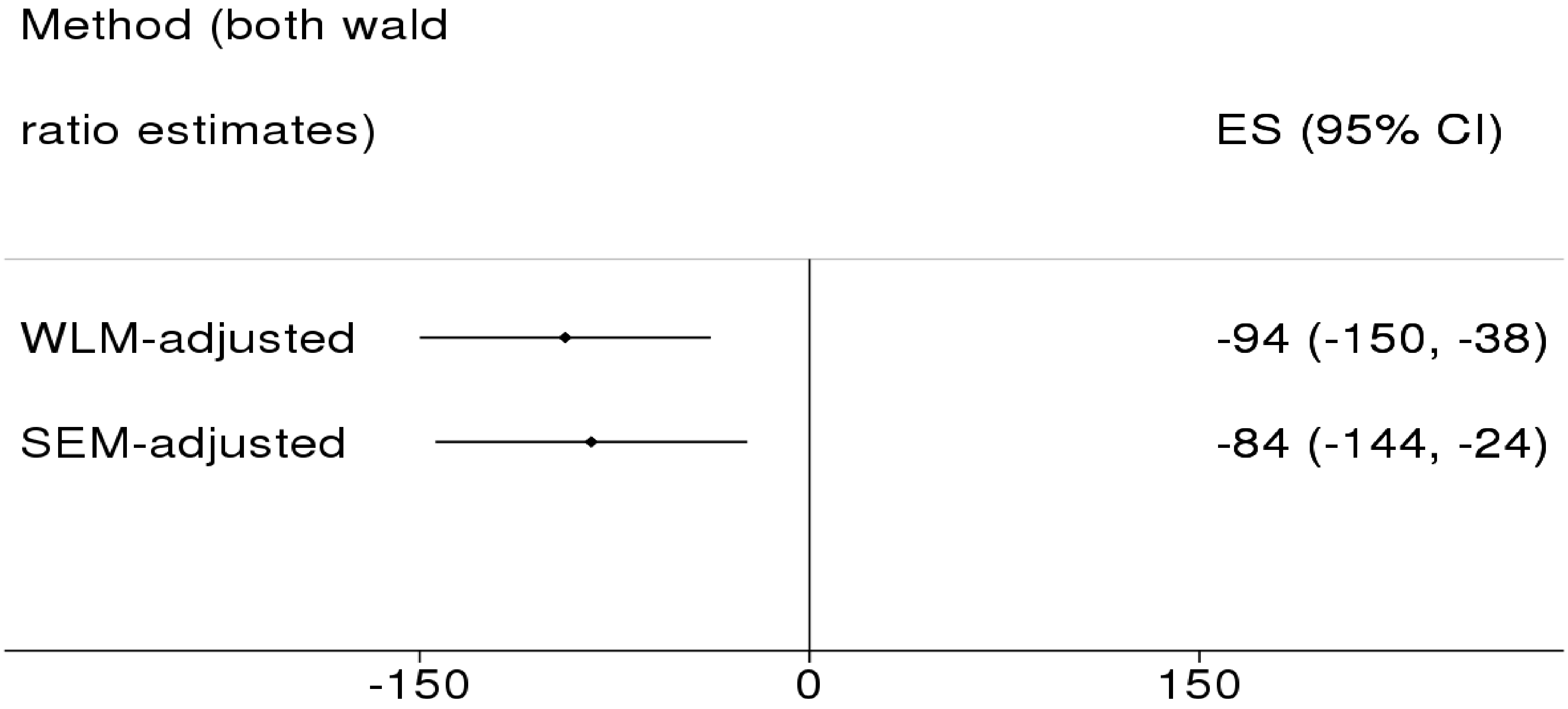
Method (both wald
ratio estimates)
ES (95% CI)
WLM-adjusted
-94 (-150, -38)
SEM-adjusted
-84 (-144, -24)
-150
0
150

ESM Figure 4: Leave-one-out analysis for the effect of genetically instrumented maternal BMI on offspring birth weight.

The x-axis shows the effect on birth weight (grams) per 1 SD increase in BMI (4 kg/m^2)

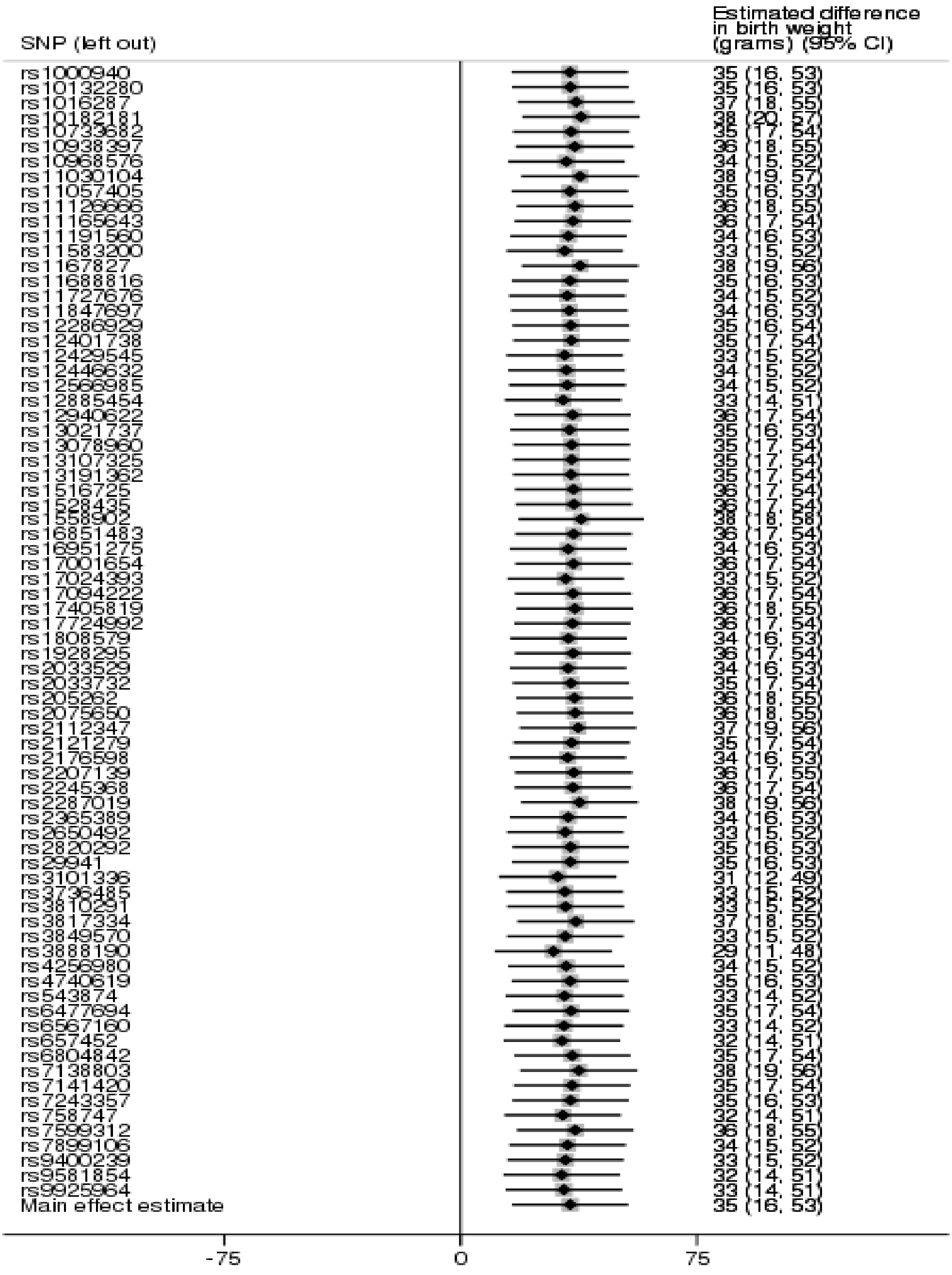

ESM Figure 5: Comparison of Wald Ratio meta-analysis estimates calculated using WLM-adjusted SNP associations from EGG + UK Biobank with estimates calculated using SEM adjusted SNP associations from UK Biobank alone, for BMI.

The x-axis shows the change in birth weight (g) per 1 SD increase in BMI (4 kg/m2), and the ES shows the estimated difference in birth weight (g) for each method.

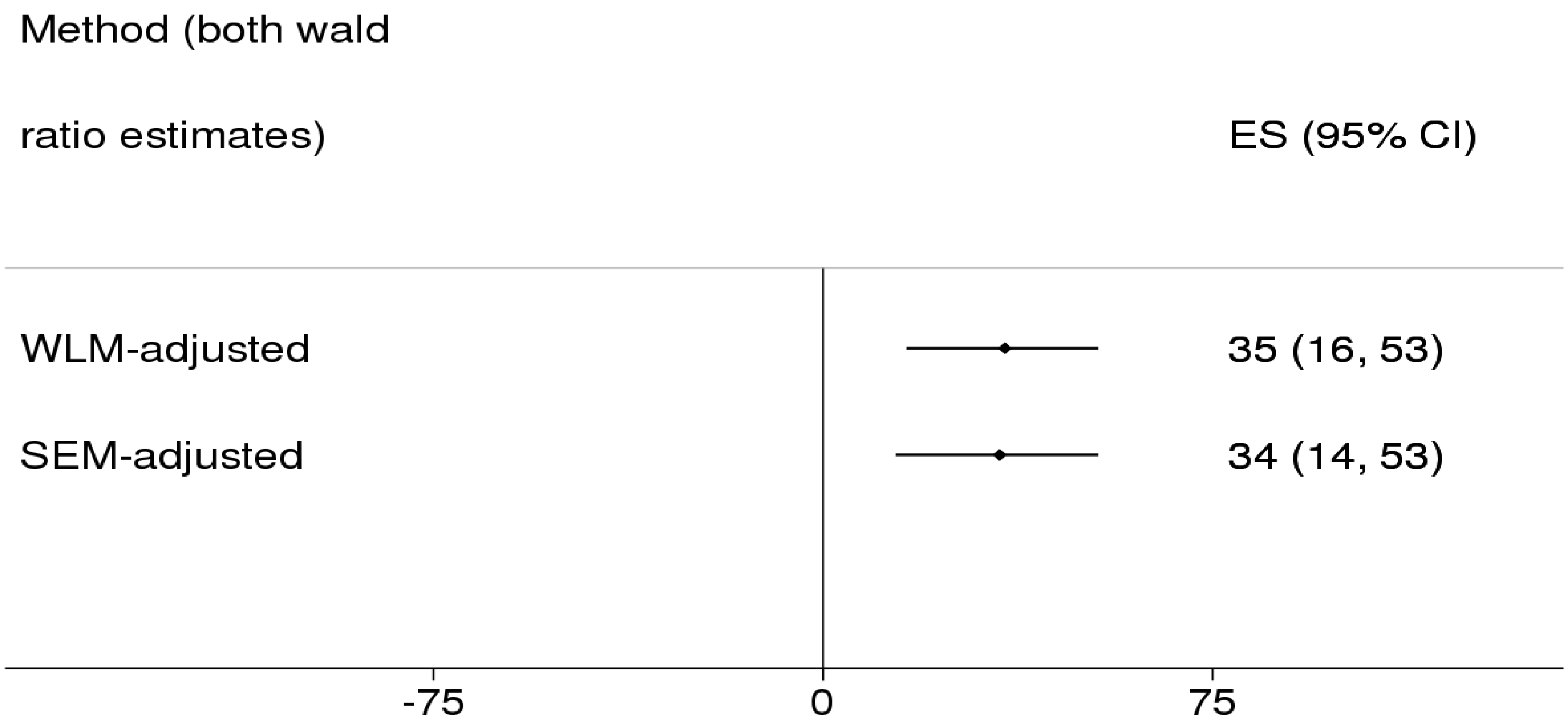
Method (both wald
ratio estimates)
ES (95% CI)
WLM-adjusted
35 (16, 53)
SEM-adjusted
34 (14, 53)
-75
0
75

ESM Figure 6: Radial MR-Egger analyses with and without outliers for metabolically favourable adiposity.

The x-axis shows the change in birth weight (g) per 1 SD increase in body fat percentage (6.5%). The radial MR-Egger estimate for the effect of maternal metabolically favourable adiposity on offspring birth weight was close to null (-2g (95% CI, -299 to 296), p = 0.99, Rücker's Q = 32.07 (d.f. = 12), I^2 = 63%, p = 0.0013), and four MR-Egger outlier SNPs were identified (**ESM Figure 7**). In an analysis with these removed the effect estimate was stronger than in the main analysis (-248g, (95% CI, -490 to -6), p = 0.046, Rücker's Q = 4.06 (d.f. = 8), I^2 = 0%, p = 0.852).

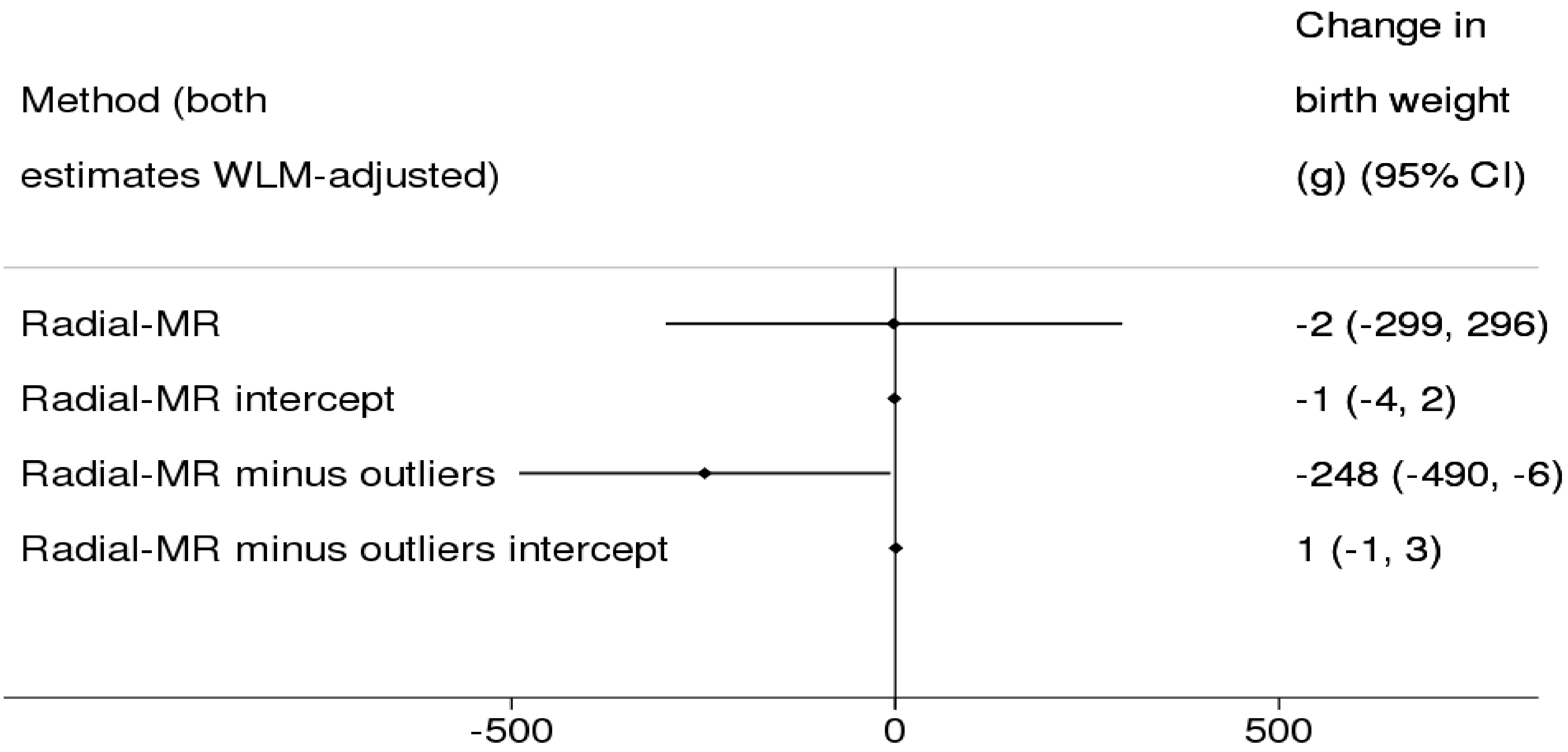
Method (both
estimates WLM-adjusted)
Change in
birth weight
(g) (95% CI)
Radial-MR
-2 (-299, 296)
Radial-MR intercept
-1 (-4, 2)
Radial-MR minus outliers
-248 (-490, -6)
Radial-MR minus outliers intercept
1 (-1, 3)
-500
0
500

ESM Figure 7: Radial MR graph for metabolically favourable adiposity.

a) The values presented are in SD units, the SD value for birth weight being 484g.

b) The confidence intervals are to 97.5%, and the results were estimated using an iterative model

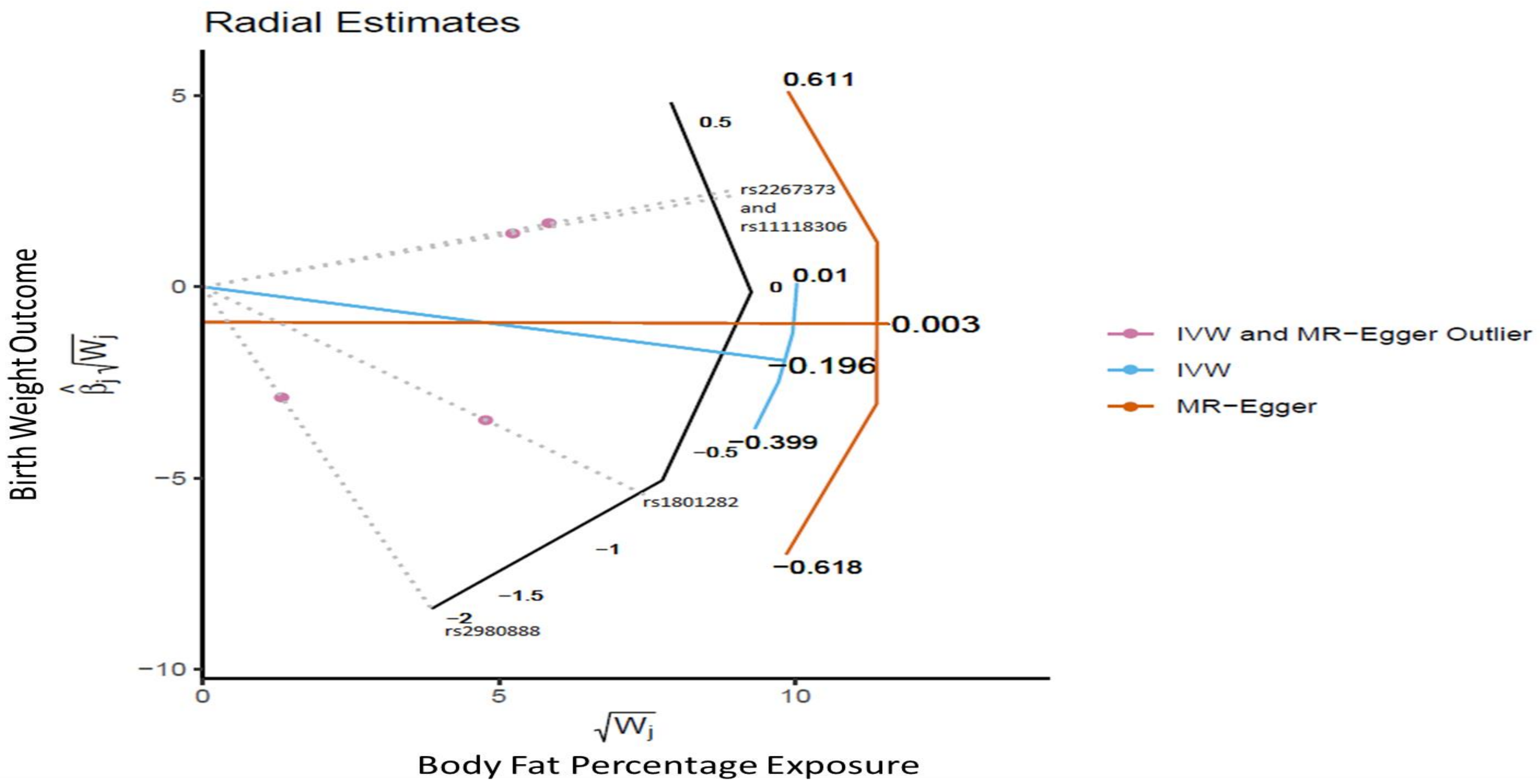

ESM Figure 8: Radial MR-Egger analyses with and without outliers for BMI.

The x-axis shows the change in birth weight (g) per 1 SD increase in BMI (4 kg/m^2). The radial MR-Egger estimate of the effect of maternal BMI on offspring birth weight was weaker (13g (95% CI, -64 to 91), p = 0.734, Rücker's Q = 177.57 (d.f. = 74), I^2 = 58%, p = 1.7e^{-10}) compared to the main estimate, and 18 outlier SNPs were identified (**ESM Figure 9**). In an analysis with these removed the effect estimate became more consistent (22g (95% CI, -32 to 77), p = 0.415, Rücker's Q = 59.77 (d.f. = 56), I^2 = 6%, p = 0.341) with the main result **(ESM Figure 8)**.

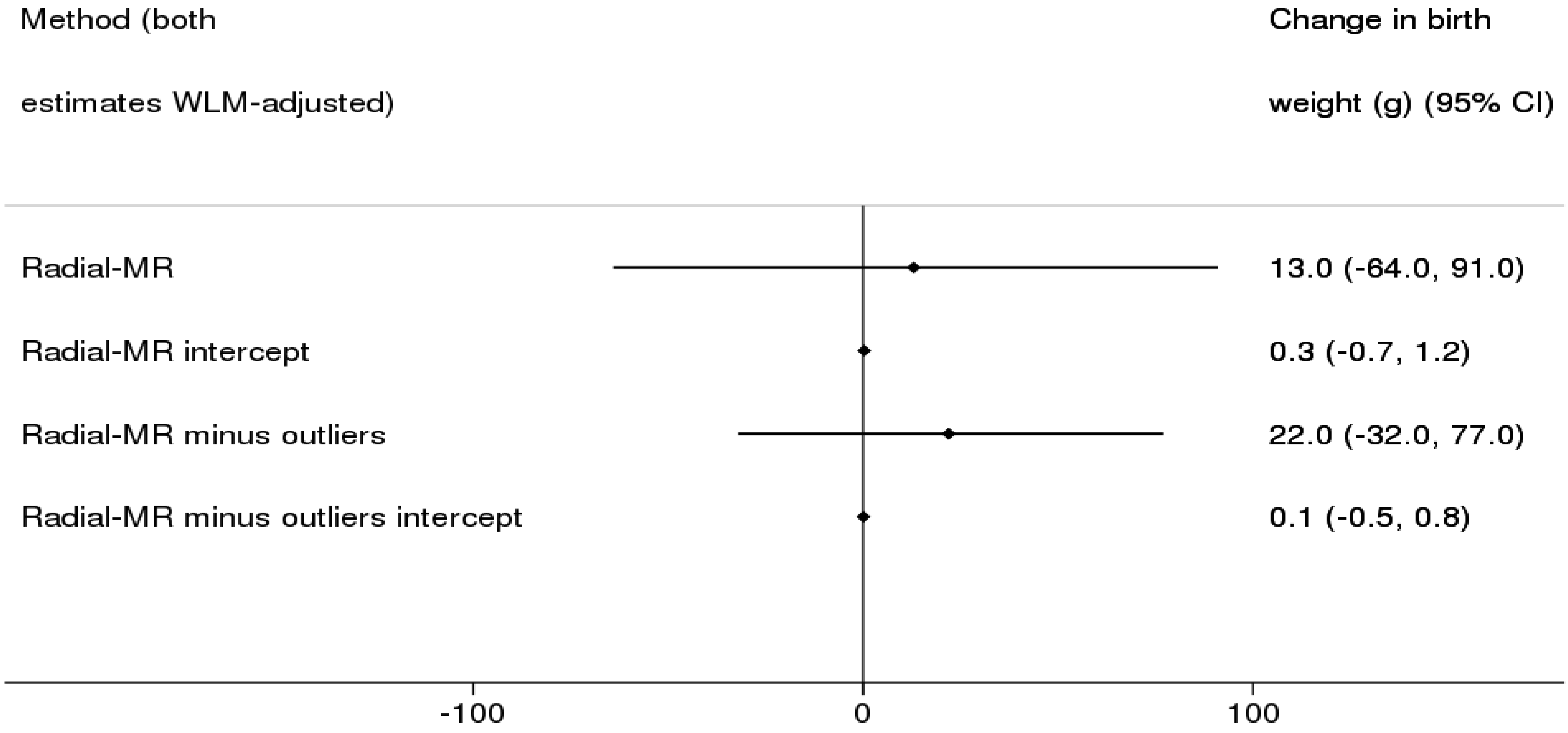
Method (both
estimates WLM-adjusted)
Change in birth
weight (g) (95% CI)
Radial-MR
13.0 (-64.0, 91.0)
Radial-MR intercept
0.3 (-0.7, 1.2)
Radial-MR minus outliers
22.0 (-32.0, 77.0)
Radial-MR minus outliers intercept
0.1 (-0.5, 0.8)
-100
0
100

ESM Figure 9: Radial MR graph for BMI.

a) The values presented are in SD units, the SD value for birth weight being 484g.
b) The confidence intervals are to 97.5%, and the results were estimated using an iterative model

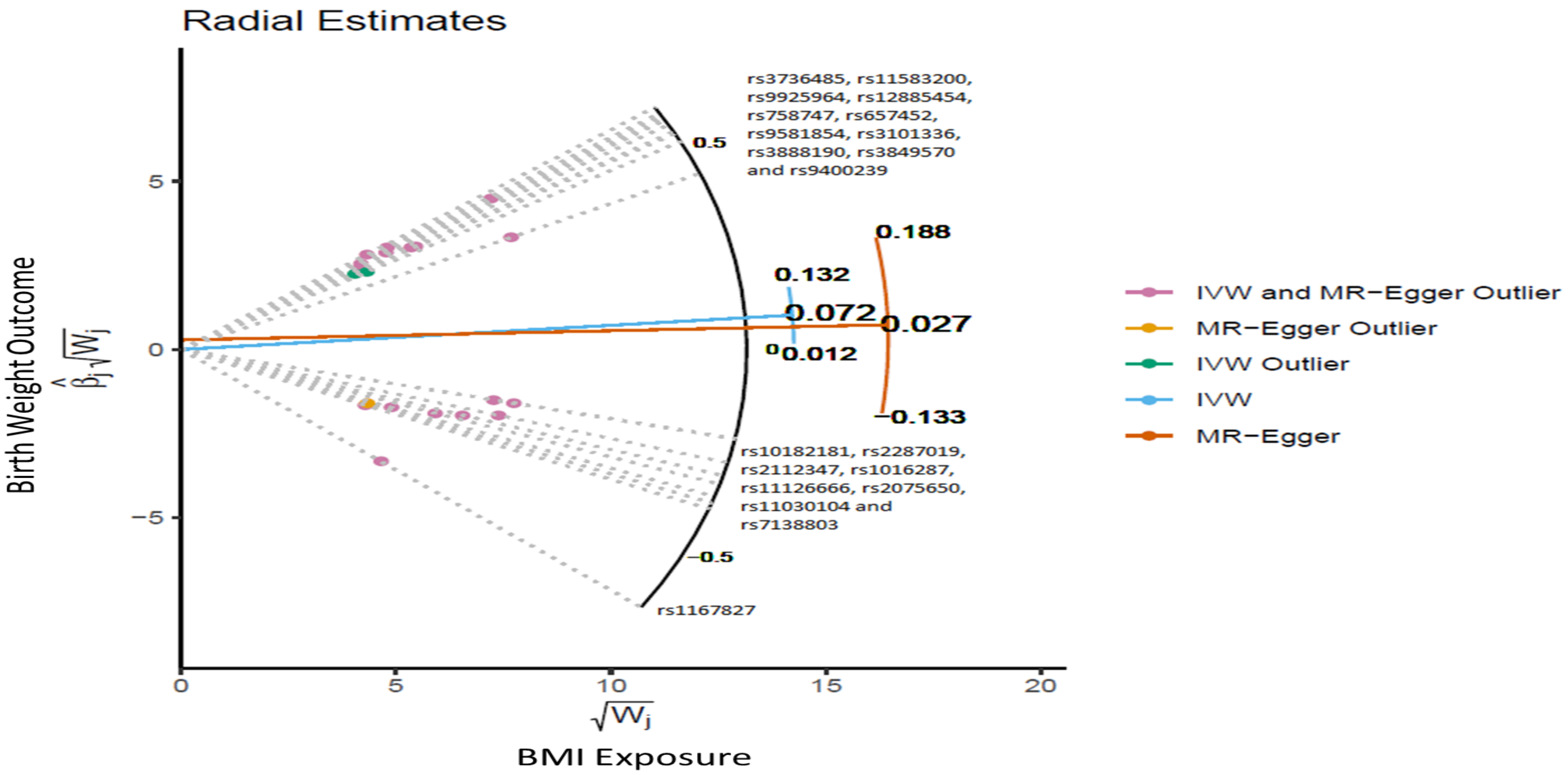

ESM Figure 10: Causative effect estimates for maternal BMI and metabolically favourable adiposity on infant cord-blood outcomes, adjusted for offspring genotype

The x-axis shows the change in infant cord-blood outcomes per 1 SD increase in maternal favourable adiposity (6.5%) and maternal BMI (4 kg/m2)

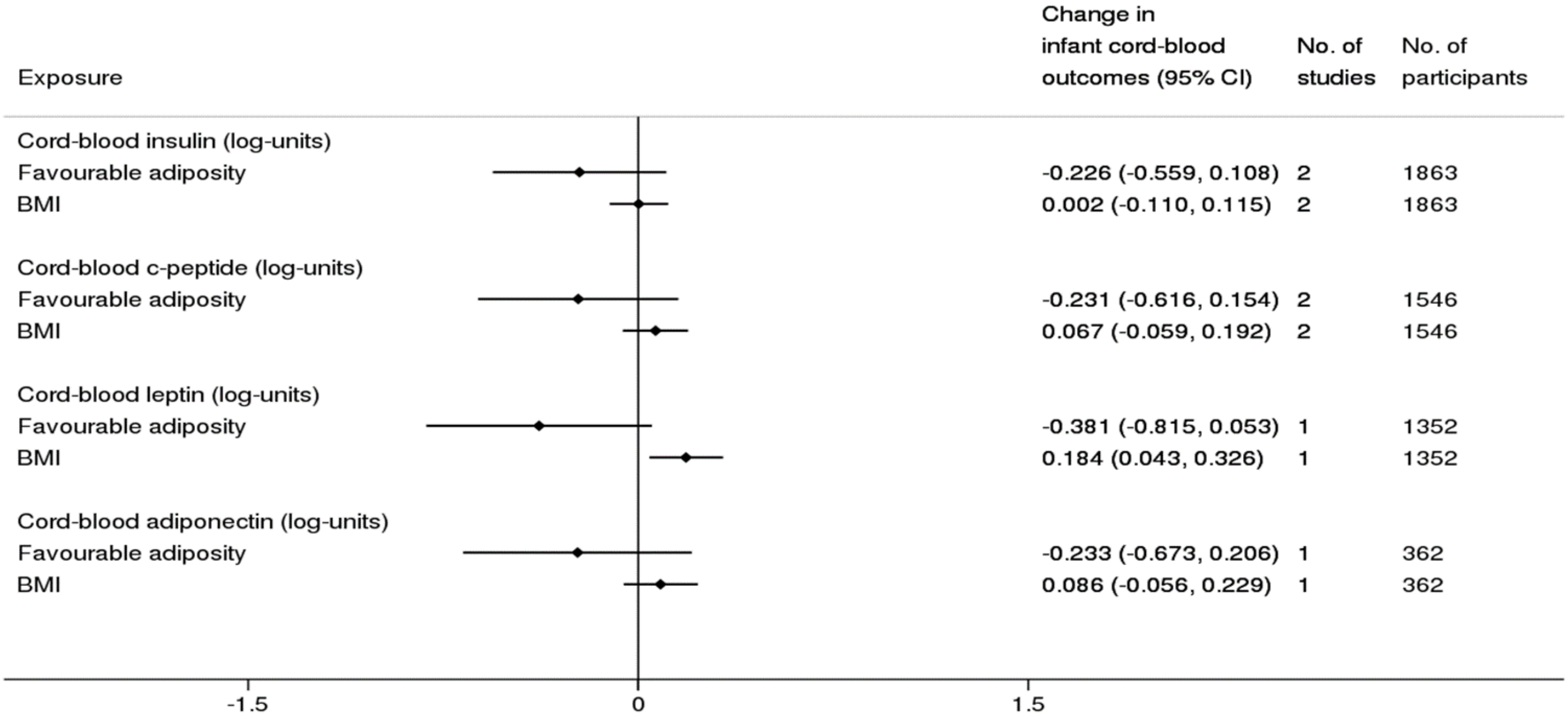

ESM Figure 11: Correlations between SNP-maternal pre-pregnancy BMI associations with SNP-GWAS BMI associations for all cohorts (in kg/m^2)

The x-axis for all of these report the SNP-BMI association as reported in Locke et al 2014 (N = 322,154). For each of the subgraphs, the y axis reports the SNP- pre-pregnancy BMI association for: all of the mother-child pair cohorts combined (N = 11,915) in a), ALSPAC (N = 6,449) in b), BiB (N = 2,853) in c), EFSOCH (N = 844) in d), HAPO 1 (N = 1,010) in e), and HAPO 2 (N = 759) in f).The green-dashed line is the predicted fit, the black line is the observed fit. As can be seen in a), though there is little predictive power (adjusted R^2=0.36) in the SNP-maternal BMI vs SNP-GWAS BMI association (likely because there is a high level of error in the associations), the size and direction of the association (1.2 ± 0.2) is consistent with a direct association. Therefore the BMI associations in the mother-child pair cohorts are valid. For each individual mother-child pair cohort, there was a consistently positive association between the SNP-maternal BMI effect and SNP-GWAS BMI effect (see b)-f)).

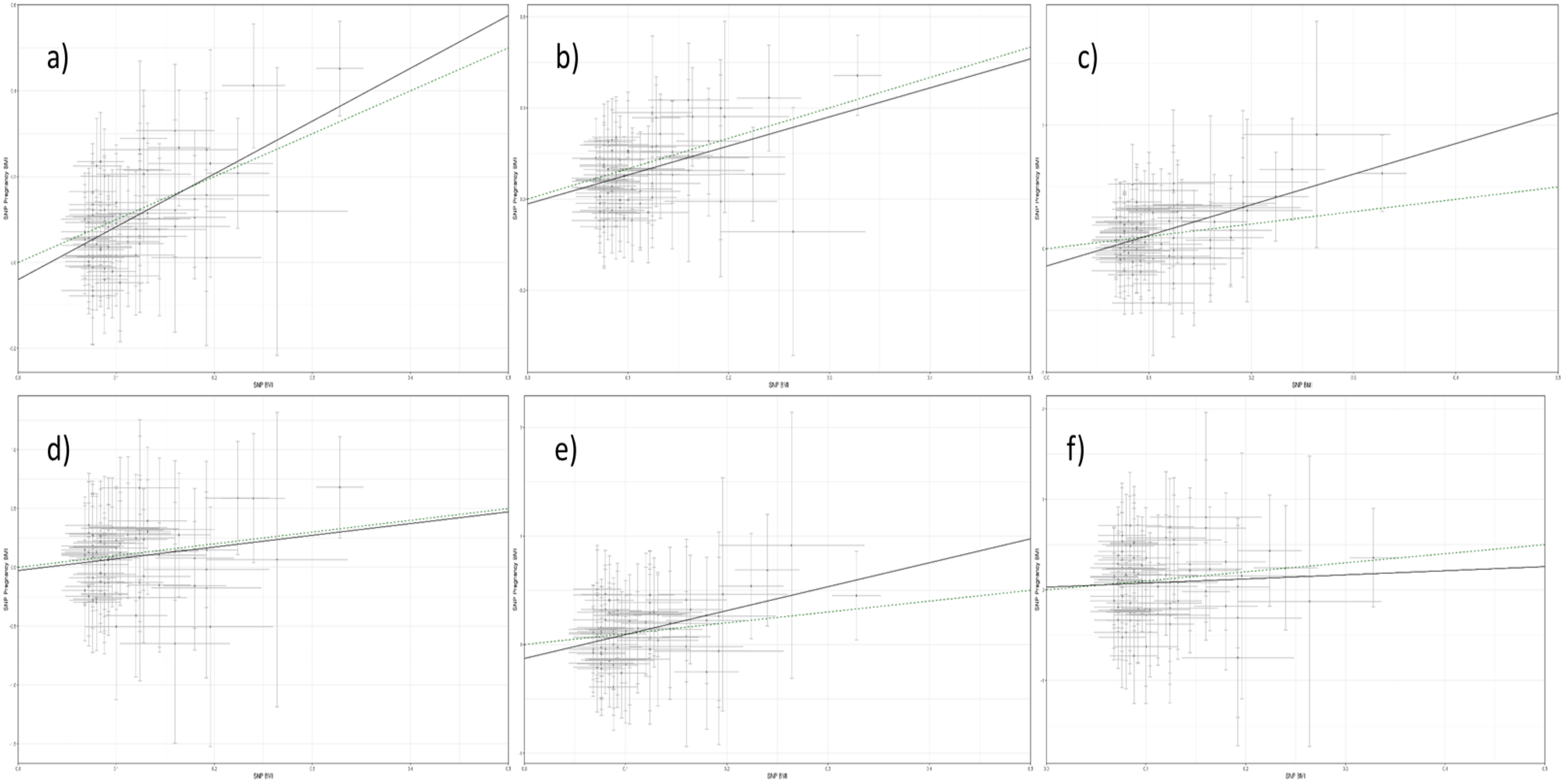
a)
b)
c)
d)
e)
f)
SNP Pregnancy BMI
SNP BMI

ESM Figure 12: Genetic associations of metabolically favourable adiposity SNPs with body fat percentage, fat mass and lean mass

The x-axis shows the effect of favourable adiposity SNPs on body mass traits (SDs) per allele. Though the metabolically favourable adiposity SNPs are associated with lower lean mass, the effect on greater fat mass and body fat percentage is eight times the magnitude (-0.0018 vs 0.0149). Thus there is no evidence of collider bias being a major factor in the association of metabolically favourable adiposity on birth weight.

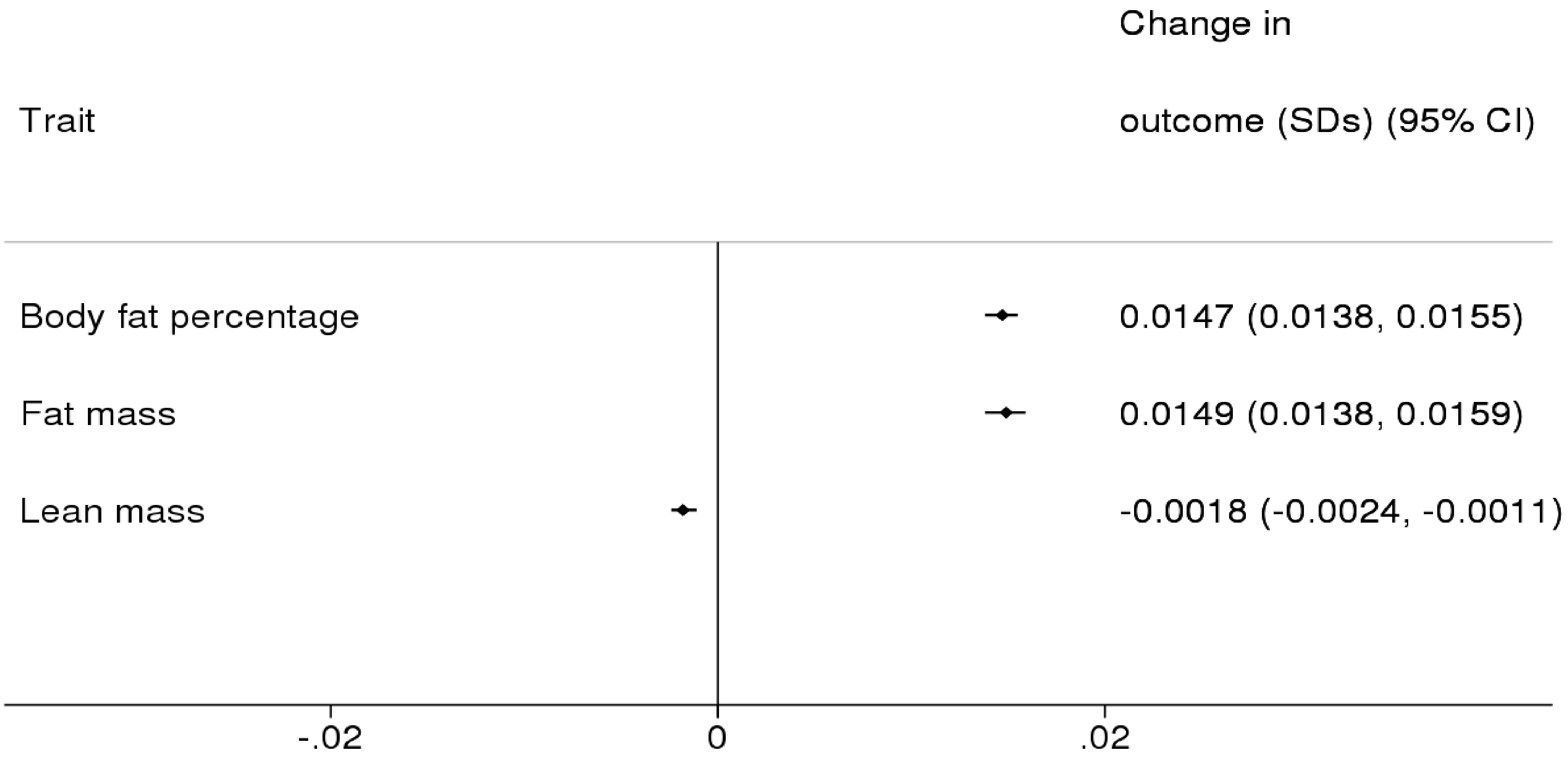
Trait
Change in
outcome (SDs) (95% CI)
Body fat percentage
0.0147 (0.0138, 0.0155)
Fat mass
0.0149 (0.0138, 0.0159)
Lean mass
-0.0018 (-0.0024, -0.0011)
-.02
0
.02

ESM Figure 13: Causative effect estimates on offspring birth weight for maternal metabolically favourable adiposity and BMI, weighted by body fat percentage

The x-axis shows the change in birth weight (g) per 1 SD increase in body fat percentage (6.5%). The effect of maternal BMI on offspring birth weight is directionally consistent with the main result when weighted by body fat percentage. Noticeably, the point estimate is larger when weighted by body fat percentage than when weighted by BMI (35g weighted by BMI vs 54g weighted by body fat percentage). This can be explained by the fact that as the SNPs are less strongly associated with the opposite exposure as they are with their primary exposure, the weighting for the opposite exposure is on average smaller, meaning a greater increase in effect on outcome per unit higher exposure.

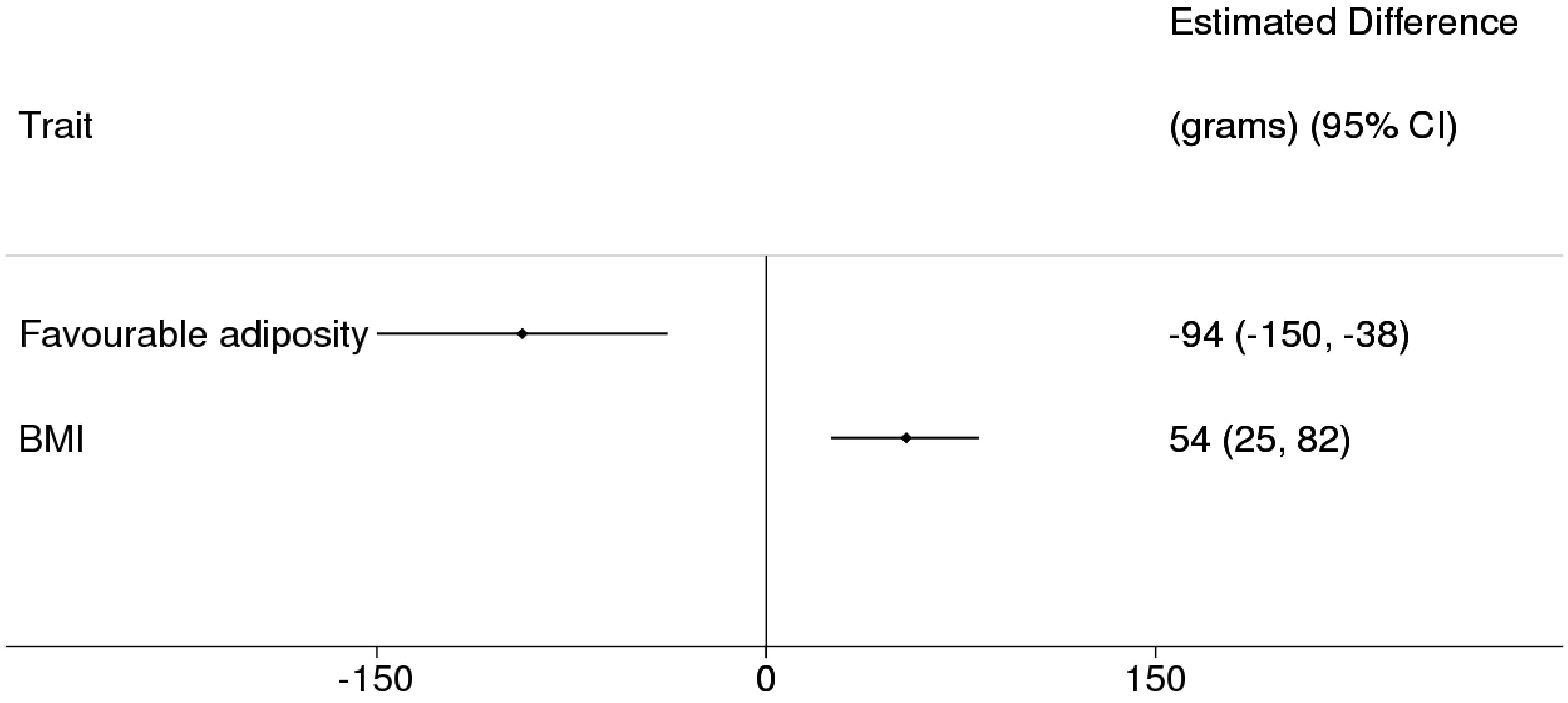
Estimated Difference
Trait
(grams) (95% CI)
Favourable adiposity
-94 (-150, -38)
BMI
54 (25, 82)
-150
0
150

ESM Figure 14: Causative effect estimates on offspring birth weight for maternal metabolically favourable adiposity and BMI, weighted by BMI

The x-axis shows the change in birth weight (g) per 1 SD increase in BMI (4 kg/m^2). The effect of maternal metabolically favourable adiposity on offspring birth weight is directionally consistent with the main result when weighted by BMI. Noticeably, the point estimate is larger when weighted by BMI than when weighted by body fat percentage (-94g weighted by body fat percentage vs -195g weighted by BMI). This can be explained by the fact that as the SNPs are less strongly associated with the opposite exposure as they are with their primary exposure, the weighting for the opposite exposure is on average smaller, meaning a greater increase in effect on outcome per unit higher exposure.

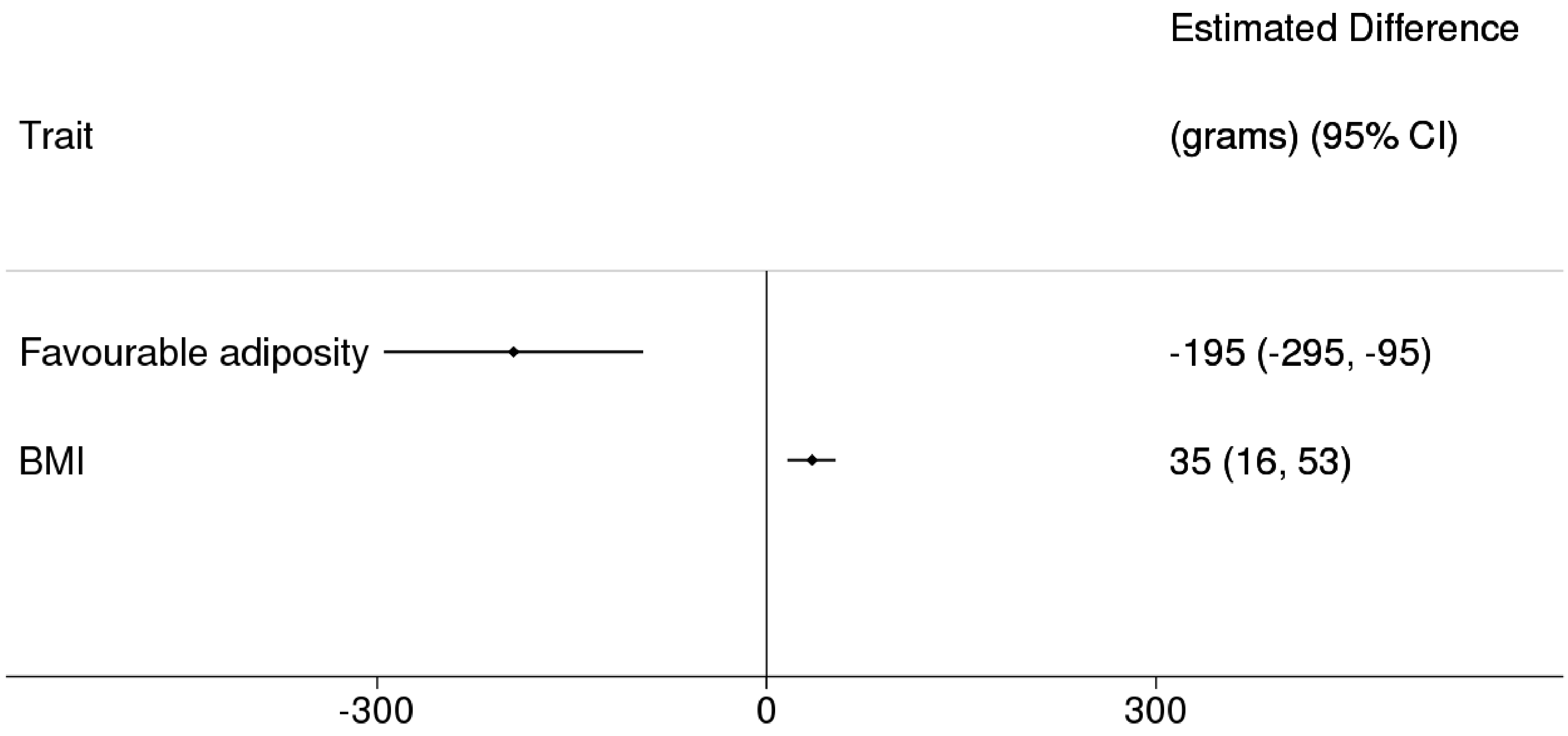
Estimated Difference
Trait
(grams) (95% CI)
Favourable adiposity
-195 (-295, -95)
BMI
35 (16, 53)
-300
0
300

References

[1] Ji Y, Yiorkas AM, Frau F, et al. (2019) Genome-Wide and Abdominal MRI Data Provide Evidence That a Genetically Determined Favorable Adiposity Phenotype Is Characterized by Lower Ectopic Liver Fat and Lower Risk of Type 2 Diabetes, Heart Disease, and Hypertension. Diabetes 68(1): 207. 10.2337/db18-0708

[2] Cichonska A, Rousu J, Marttinen P, et al. (2016) metaCCA: summary statistics-based multivariate meta-analysis of genome-wide association studies using canonical correlation analysis. Bioinformatics 32(13): 1981-1989. 10.1093/bioinformatics/btw052

[3] Hewitt J, Walters M, Padmanabhan S, Dawson J (2016) Cohort profile of the UK Biobank: diagnosis and characteristics of cerebrovascular disease. BMJ Open 6(3)

[4] Boyd A, Golding J, Macleod J, et al. (2013) Cohort Profile: The 'Children of the 90s'—the index offspring of the Avon Longitudinal Study of Parents and Children. International Journal of Epidemiology 42(1): 111-127. 10.1093/ije/dys064

[5] Fraser A, Macdonald-Wallis C, Tilling K, et al. (2013) Cohort Profile: The Avon Longitudinal Study of Parents and Children: ALSPAC mothers cohort. International Journal of Epidemiology 42(1): 97-110. 10.1093/ije/dys066

[6] (2002-2017) Explore data and samples. Available from http://www.bristol.ac.uk/alspac/researchers/our-data/. Accessed 16th of August 2018

[7] Wright J, Small N, Raynor P, et al. (2012) Cohort Profile: The Born in Bradford multi-ethnic family cohort study. International Journal of Epidemiology 42(4): 978-991. 10.1093/ije/dys112

[8] Knight B, Shields BM, Hattersley AT (2006) The Exeter Family Study of Childhood Health (EFSOCH): study protocol and methodology. Paediatric and Perinatal Epidemiology 20(2): 172-179. 10.1111/j.1365-3016.2006.00701.x

[9] (2008) Hyperglycemia and Adverse Pregnancy Outcomes. New England Journal of Medicine 358(19): 1991-2002. 10.1056/NEJMoa0707943

[10] Warrington NM, Beaumont RN, Horikoshi M, et al. (2019) Maternal and fetal genetic effects on birth weight and their relevance to cardio-metabolic risk factors. Nature Genetics 51(5): 804-814. 10.1038/s41588-019-0403-1

[11] Abraham G, Inouye M (2014) Fast Principal Component Analysis of Large-Scale Genome-Wide Data. PLOS ONE 9(4): e93766. 10.1371/journal.pone.0093766

[12] Cummins C, Winter H, Cheng KK, Maric R, Silcocks P, Varghese C (1999) An assessment of the Nam Pehchan computer program for the identification of names of south Asian ethnic origin. Journal of public health medicine 21(4): 401-406

[13] Bycroft C, Freeman C, Petkova D, et al. (2018) The UK Biobank resource with deep phenotyping and genomic data. Nature 562(7726): 203-209. 10.1038/s41586-018-0579-z

[14] UKBiobank, theAccessTeam (2017) Important note about imputed genetics data. Available from http://www.ukbiobank.ac.uk/2017/07/important-note-about-imputed-genetics-data/. Accessed 1st of March 2018

[15] Loh P-R, Tucker G, Bulik-Sullivan BK, et al. (2015) Efficient Bayesian mixed model analysis increases association power in large cohorts. Nature genetics 47(3): 284-290. 10.1038/ng.3190

[16] THE BORN IN BRADFORD PLATFORM. Available from https://borninbradford.nhs.uk/wp-content/uploads/BiB_Data_Summary_slides.pdf. Accessed 26th of March 2019

[17] Hughes AE, Nodzenski M, Beaumont RN, et al. (2018) Fetal Genotype and Maternal Glucose Have Independent and Additive Effects on Birth Weight. Diabetes 67(5): 1024. 10.2337/db17-1188
[18] Zhao Y, Wang SF, Mu M, Sheng J (2012) Birth weight and overweight/obesity in adults: a meta-analysis. European journal of pediatrics 171(12): 1737-1746. 10.1007/s00431-012-1701-0
[19] Dupuis J, Langenberg C, Prokopenko I, et al. (2010) New genetic loci implicated in fasting glucose homeostasis and their impact on type 2 diabetes risk. Nature genetics 42(2): 105-116. 10.1038/ng.520
[20] Hartwig FP, Davies NM, Hemani G, Davey Smith G (2016) Two-sample Mendelian randomization: avoiding the downsides of a powerful, widely applicable but potentially fallible technique. International Journal of Epidemiology 45(6): 1717-1726. 10.1093/ije/dyx028
[21] Lawlor DA, West J, Fairley L, et al. (2014) Pregnancy glycaemia and cord-blood levels of insulin and leptin in Pakistani and white British mother-offspring pairs: findings from a prospective pregnancy cohort. Diabetologia 57(12): 2492-2500. 10.1007/s00125-014-3386-6
[22] Shields BM, Knight B, Hopper H, et al. (2007) Measurement of Cord Insulin and Insulin-Related Peptides Suggests That Girls Are More Insulin Resistant Than Boys at Birth. Diabetes Care 30(10): 2661. 10.2337/dc06-1501
[23] Nesbitt GS, Smye M, Sheridan B, Lappin TRJ, Trimble ER (2006) Integration of local and central laboratory functions in a worldwide multicentre study: Experience from the Hyperglycemia and Adverse Pregnancy Outcome (HAPO) Study. Clinical Trials 3(4): 397-407. 10.1177/1740774506070695
[24] Farrar D, Fairley L, Santorelli G, et al. (2015) Association between hyperglycaemia and adverse perinatal outcomes in south Asian and white British women: analysis of data from the Born in Bradford cohort. The lancet Diabetes & endocrinology 3(10): 795-804. 10.1016/s2213-8587(15)00255-7
[25] Weedon MN, Frayling TM, Shields B, et al. (2005) Genetic Regulation of Birth Weight and Fasting Glucose by a Common Polymorphism in the Islet Cell Promoter of the Glucokinase Gene. Diabetes 54(2): 576. 10.2337/diabetes.54.2.576
[26] Lowe LP, Metzger BE, Dyer AR, et al. (2012) Hyperglycemia and Adverse Pregnancy Outcome (HAPO) Study: associations of maternal A1C and glucose with pregnancy outcomes. Diabetes care 35(3): 574-580. 10.2337/dc11-1687
[27] Lawlor D, Richmond R, Warrington N, et al. (2017) Using Mendelian randomization to determine causal effects of maternal pregnancy (intrauterine) exposures on offspring outcomes: Sources of bias and methods for assessing them. Wellcome open research 2: 11-11. 10.12688/wellcomeopenres.10567.1
[28] Warrington NM, Freathy RM, Neale MC, Evans DM (2018) Using structural equation modelling to jointly estimate maternal and fetal effects on birthweight in the UK Biobank. International Journal of Epidemiology: dyy015-dyy015. 10.1093/ije/dyy015
[29] Lu Y, Day FR, Gustafsson S, et al. (2016) New loci for body fat percentage reveal link between adiposity and cardiometabolic disease risk. Nature Communications 7: 10495. 10.1038/ncomms10495

https://www.nature.com/articles/ncomms10495#supplementary-information
[30] Tyrrell J, Richmond RC, Palmer TM, et al. (2016) Genetic evidence for causal relationships between maternal obesity-related traits and birth weight. Jama 315(11): 1129-1140. 10.1001/jama.2016.1975
[31] Steichen T (2001) METANINF: Stata Module to Evaluate Influence of a Single Study in Meta-analysis Estimation
[32] Bowden J, Davey Smith G, Burgess S (2015) Mendelian randomization with invalid instruments: effect estimation and bias detection through Egger regression. International Journal of Epidemiology 44(2): 512-525. 10.1093/ije/dyv080

[33] Bowden J, Davey Smith G, Haycock PC, Burgess S (2016) Consistent Estimation in Mendelian Randomization with Some Invalid Instruments Using a Weighted Median Estimator. Genetic Epidemiology 40(4): 304-314. 10.1002/gepi.21965
[34] Davey Smith G, Spiller W, Bowden J, et al. (2018) Improving the visualization, interpretation and analysis of two-sample summary data Mendelian randomization via the Radial plot and Radial regression. International Journal of Epidemiology 47(4): 1264-1278. 10.1093/ije/dyy101
[35] Hartwig FP, Tilling K, Davey Smith G, Lawlor DA, Borges MC (2019) Bias in two-sample Mendelian randomization by using covariable-adjusted summary associations. bioRxiv: 816363. 10.1101/816363
[36] Aschard H, Vilhjálmsson Bjarni J, Joshi Amit D, Price Alkes L, Kraft P (2015) Adjusting for Heritable Covariates Can Bias Effect Estimates in Genome-Wide Association Studies. The American Journal of Human Genetics 96(2): 329-339. https://doi.org/10.1016/j.ajhg.2014.12.021
[37] Locke AE, Kahali B, Berndt SI, et al. (2015) Genetic studies of body mass index yield new insights for obesity biology. Nature 518: 197. 10.1038/nature14177

https://www.nature.com/articles/nature14177#supplementary-information
[38] Yang Q, Sanderson E, Tilling K, Borges MC, Lawlor DA (2019) Exploring and mitigating potential bias when genetic instrumental variables are associated with multiple non-exposure traits in Mendelian randomization. medRxiv: 19009605. 10.1101/19009605
[39] Brand JS, Gaillard R, West J, et al. (2019) Associations of maternal quitting, reducing, and continuing smoking during pregnancy with longitudinal fetal growth: Findings from Mendelian randomization and parental negative control studies. PLoS medicine 16(11): e1002972. 10.1371/journal.pmed.1002972
[40] Freathy RM, Kazeem GR, Morris RW, et al. (2011) Genetic variation at CHRNA5-CHRNA3-CHRNB4 interacts with smoking status to influence body mass index. Int J Epidemiol 40(6): 1617-1628. 10.1093/ije/dyr077
[41] The T, Genetics C, Furberg H, et al. (2010) Genome-wide meta-analyses identify multiple loci associated with smoking behavior. Nature Genetics 42: 441. 10.1038/ng.571

https://www.nature.com/articles/ng.571#supplementary-information
[42] Okbay A, Beauchamp JP, Fontana MA, et al. (2016) Genome-wide association study identifies 74 loci associated with educational attainment. Nature 533: 539. 10.1038/nature17671
[43] Qingyuan Z, Jingshu W, Gibran H, Jack B, Dylan SS (2020) Statistical inference in two-sample summary-data Mendelian randomization using robust adjusted profile score. The Annals of Statistics 48(3): 1742-1769. 10.1214/19-AOS1866
